# Parallel body shape divergence in the Neotropical fish genus *Rhoadsia* (Teleostei: Characidae) along elevational gradients of the western slopes of the Ecuadorian Andes

**DOI:** 10.1371/journal.pone.0179432

**Published:** 2017-06-28

**Authors:** Grace Malato, Virginia R. Shervette, Ronald Navarrete Amaya, Jonathan Valdiviezo Rivera, Fredy Nugra Salazar, Paola Calle Delgado, Kirby C. Karpan, Windsor E. Aguirre

**Affiliations:** 1 Department of Biological Sciences, DePaul University, Chicago, Illinois, United States of America; 2 Department of Biology/Geology, University of South Carolina, Aiken, South Carolina, United States of America; 3 Urbanización Paraíso del Río 1, Guayaquil, Ecuador; 4 Museo de Ciencias Naturales del Instituto Nacional de Biodiversidad, Quito, Ecuador; 5 Laboratorio de Zoología de Vertebrados de la Universidad del Azuay, Cuenca, Ecuador; 6 Facultad de Ciencias de la Vida, Escuela Superior Politécnica del Litoral, Guayaquil, Ecuador; SOUTHWEST UNIVERSITY, CHINA

## Abstract

Neotropical mountain streams are important contributors of biological diversity. Two species of the characid genus *Rhoadsia* differing for an ecologically important morphological trait, body depth, have been described from mountain streams of the western slopes of the Andes in Ecuador. *Rhoadsia altipinna* is a deeper-bodied species reported from low elevations in southwestern Ecuador and northern Peru, and *Rhoadsia minor* is a more streamlined species that was described from high elevations (>1200 m) in the Esmeraldas drainage in northwestern Ecuador. Little is known about these species and their validity as distinct species has been questioned. In this study, we examine how their body shape varies along replicated elevational gradients in different drainages of western Ecuador using geometric morphometrics and the fineness ratio. We also use sequences of the mitochondrial cytochrome oxidase c I gene and the second intron of the S7 nuclear gene to examine whether genetic data are consistent with the existence of two species. We found that body depth varies continuously among populations within drainages as a function of elevation, and that body shape overlaps among drainages, such that low elevation populations of *R*. *minor* in the Esmeraldas drainage have similar body depths to higher elevation *R*. *altipinna* in southern drainages. Although a common general trend of declining body depth with elevation is clear, the pattern and magnitude of body shape divergence differed among drainages. Sequencing of mitochondrial and nuclear genes failed to meet strict criteria for the recognition of two species (e.g., reciprocal monophyly and deep genetic structure). However, there was a large component of genetic variation for the COI gene that segregated among drainages, indicating significant genetic divergence associated with geographic isolation. Continued research on *Rhoadsia* in western Ecuador may yield significant insight into adaptation and speciation in Neotropical mountain streams.

## Introduction

Habitat heterogeneity is a crucial factor promoting the evolution of biological diversity [1–3]. As the characteristics of the available habitats change, so do organismal fitness functions resulting in selection in favor of different suites of traits in different habitats [4] and the evolution of phenotypically divergent populations [5–8]. These populations may eventually evolve reproductive isolation and become distinct species [2, 9]. Adaptive evolution is not the only mechanism responsible for the phenotypic divergence of geographically isolated populations. Genetic drift, a process that is especially important in small populations, can also lead to genetically based non-adaptive phenotypic divergence [10–12], as can phenotypic plasticity, phenotypic divergence resulting from environmental variation and not DNA sequence changes [13, 14].

Mountain streams in Neotropical regions offer tremendous potential for studying evolution and speciation in some of the most biodiverse ecosystems on Earth. Not only do particular stream tracts often exhibit substantial habitat complexity, allowing coexistence of closely related species through niche partitioning [15–17], but habitat conditions can change rapidly with elevation across streams in a predictable fashion [18, 19]. Among the abiotic factors that covary with elevation are temperature, oxygen concentration, turbidity, water velocity, sediment particle size, energy input sources, and nutrient levels [19, 20]. Biological communities also change in predictable ways, with well-documented patterns including changes in species diversity [20, 21] and the proportions of species of different functional/ taxonomic groups [20, 22, 23]. In cases in which mountains are close to the ocean, like the Andes along the Pacific coast of South America, there may be a significant number of geographically neighboring rivers that run independently into the ocean, forming naturally replicated ecosystems with similar geological histories. Western Ecuador is one such area.

**Fig 1:**
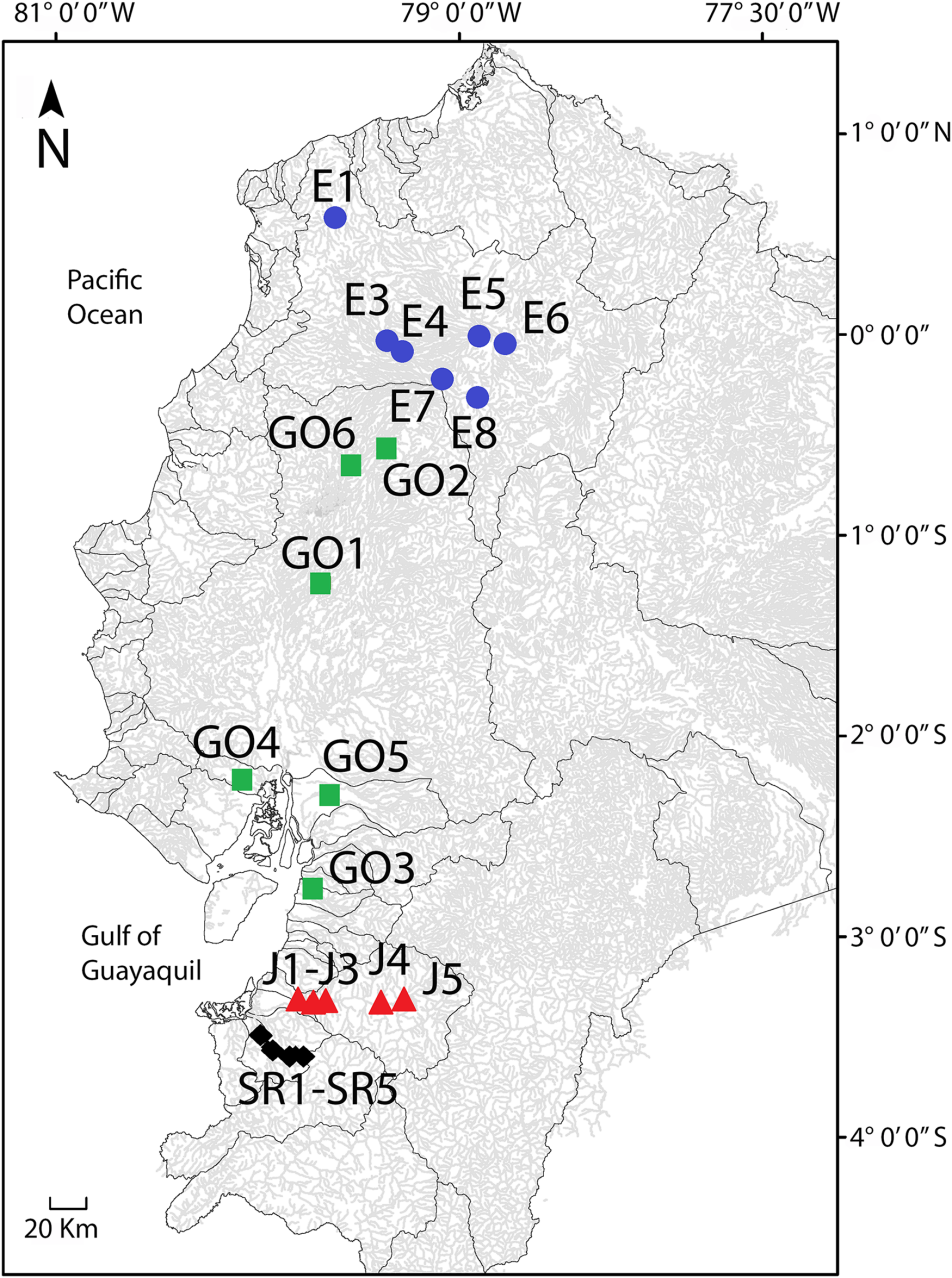
Map of sampling sites in western Ecuador with major drainage systems outlined. From north E = Esmeraldas drainage, GO = Guayas and other small neighboring drainages, J = Jubones, and SR = Santa Rosa.

Ecuador is a country that is strongly influenced by the Andes Mountains, which bisect the low-elevation areas into western and eastern regions. The eastern region includes part of the headwaters of the Amazon River and possesses some of the most biodiverse ecosystems on Earth [24, 25]. Species diversity is lower in the western region but the rates of endemism of freshwater fishes are among the highest in the Neotropics [26–28]. Despite the high species endemism, the fishes in the area belong to the same major fish groups that dominate larger and more diverse Neotropical rivers in other regions [26]. Rivers in western Ecuador run relatively short distances between the Andes and the Pacific Ocean, forming a series of independent drainage systems (Fig 1), each harboring generally similar fish communities that shift along elevational gradients as environmental conditions change [28]. These present an opportunity to study how Neotropical stream fishes adapt to, and diversify along, elevational gradients in Neotropical mountain streams. In addition, while Ecuador as a whole harbors approximately 824 described freshwater fish species, only 112 of these occur west of the Andes and 70 or less species have been recorded from any given drainage [28]. The relatively low fish species diversity in western Ecuador should theoretically provide greater ecological opportunity for intraspecific diversification through reduced interspecific competition [9, 29, 30] facilitating studies aimed at deciphering the early stages of the process of adaptation and speciation. *Rhoadsia* is a characid fish genus in western Ecuador that we think may exhibit significant potential in this regard.

**Fig 2:**
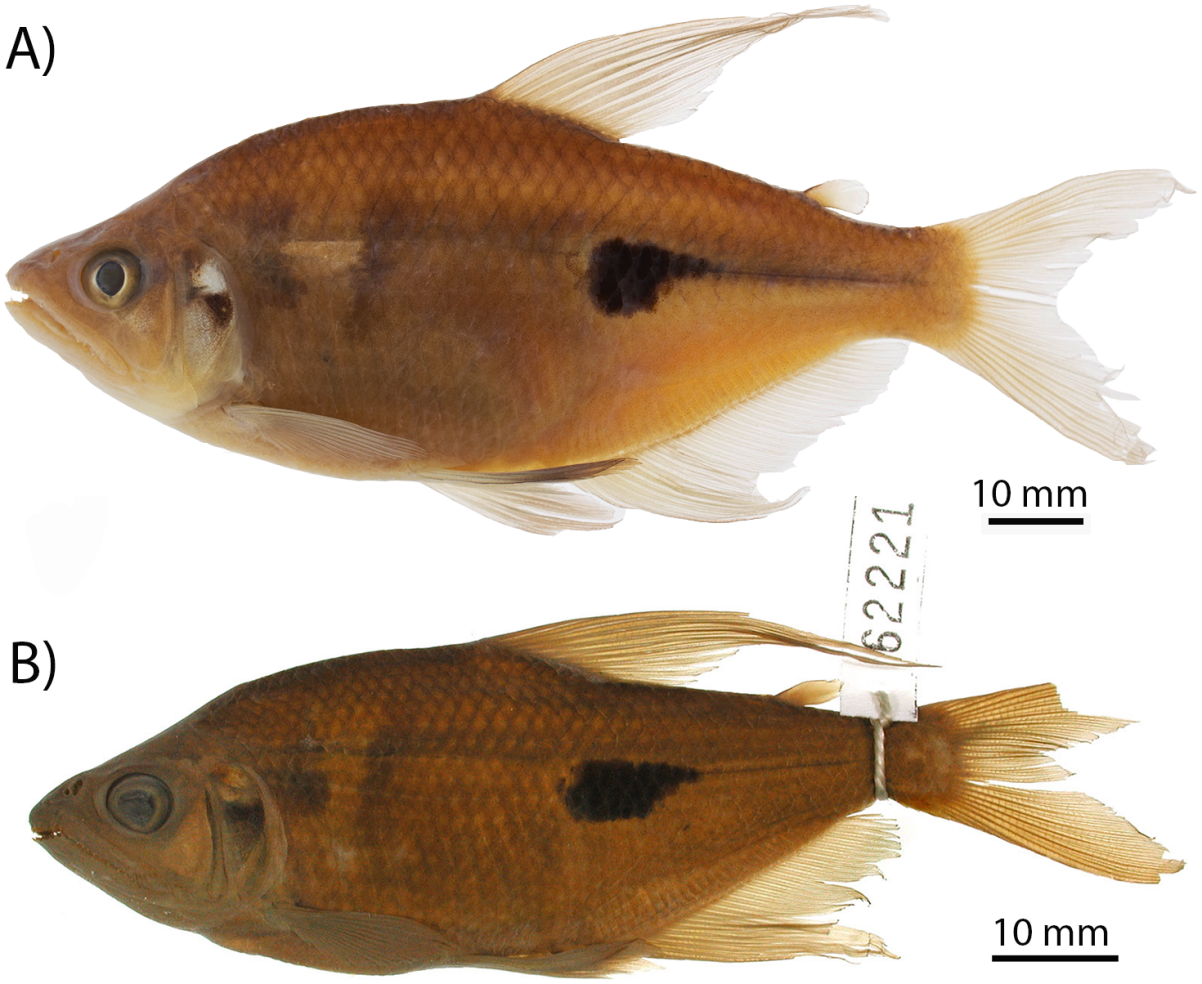
*Rhoadsia* spp. from western Ecuador. A) Representative specimen of *R*. *altipinna* from the Guayas River drainage (Río Palenque, FMNH-79080). B) Holotype of *R*. *minor* from Mindo in the upper Esmeraldas drainage (CAS-62221).

The genus *Rhoadsia* is endemic to western Ecuador and northwestern Peru and belongs to the Rhoadsiinae, a small, morphologically distinctive subfamily within the hyper-diverse Characidae [31]. *Rhoadsia* is the only genus in the Rhoadsiinae present in Ecuador, making it easy to identify, with other genera replacing it northward in western Colombia and Central America [32, 33]. Despite its relatively restricted geographic range, *Rhoadsia* is abundant in streams throughout western Ecuador and occurs over a relatively broad elevational range from sea level to approximately 1300 m above sea level [34, 35]. It is highly divergent morphologically from all other characids in western Ecuador, having a much deeper body and a distinctive color pattern [34, 36]. The difference in body depth relative to other characids is particularly likely to be important ecologically since body depth is known to be an adaptively important trait across fishes [37]. *Rhoadsia* also exhibits substantial sexual dimorphism, with males growing much larger than females, and exhibiting bright breeding colors, greatly extended dorsal and anal fin rays, and larger mouths than females [36]. It is worth noting that this sexual dimorphism develops with size, small to intermediate size males generally resemble females in body shape [36]. Two species of *Rhoadsia* are presently recognized in western Ecuador (Fig 2). *Rhoadsia altipinna* is a larger, deeper-bodied species reported from low elevations in southwestern Ecuador and northern Peru, and *Rhoadsia minor* is a smaller, more streamlined species that was described from high elevations (>1200 m) in the Esmeraldas drainage in northwestern Ecuador [27, 28, 34, 35, 38]. Other minor differences recorded between species include a more forward placement of the dorsal fin, a relatively longer maxillary with feebler teeth, and a smaller size at sexual maturity in *R*. *minor* [35].

Only a few published studies on *Rhoadsia* exist [28, 39, 40]. Despite their present status, there appears to be some morphological overlap between the two species [34], such that the status of *R*. *minor* as a valid species has been questioned [41]. The precise distribution of *R*. *minor* is also not clear since the type locality is at high elevation (1260 m) but presently all *Rhoadsia* in the Esmeraldas drainage, including populations at low elevations, are listed as *R*. *minor* [27, 28], despite some evidence that these low elevation populations may be more similar morphologically to low elevation *R*. *altipinna* [34]. Furthermore, in a survey of *R*. *altipinna* collected at five sites between 31 and 613 m in elevation in the Santa Rosa River in southwestern Ecuador, Aguirre et al. [36] found that body depth declined significantly with elevation, suggesting that the primary characteristic used to distinguish the two species covaries with elevation.

The main goal of this study is to fill some of the gaps in knowledge of *Rhoadsia* spp. in western Ecuador. Specifically, we examine how body shape varies in *Rhoadsia* spp. along replicated elevational gradients and compare patterns of variation among rivers, substantially expanding the elevational and geographic ranges previously evaluated. We use geometric morphometrics [42] to examine general body shape divergence and also use the Fineness Ratio (FR = standard length / body depth), a more traditional measure of body elongation that has been used to describe body depth divergence between the two species in the past [34, 38]. We also examine whether genetic data are consistent with the existence of two species of *Rhoadsia* in the study area by sequencing the mitochondrial cytochrome oxidase c I (COI) gene and a fragment from the second intron of the S7 nuclear gene (S7i2) for subsets of specimens from throughout the study area. The COI gene was selected because it is the most common DNA barcoding gene used to identify metazoan species [43]. The S7i2 gene was used because it is a nuclear genome marker allowing inference beyond the mitochondrial genome, has a relatively high rate of evolution for a nuclear marker because it is non-coding, and is one of the most commonly used nuclear markers in studies of fish phylogeography [44–47].

## Materials and methods

### Ethics statement

All procedures were conducted in accordance with the guidelines of the National Research Council of the National Academies of the United States of America and approved by the DePaul University Institutional Animal Care and Use Committee (protocol # 14–002). Field research was conducted under permit # 013–14 IC-FAU-DNB/MA from the Ministry of the Environment of Ecuador and complied with all relevant regulations. Specimens were euthanized immediately after collection using buffered tricaine methanesulfonate, MS-222, pH 7.5 in water until 10 min after opercular movement stopped. No experimental procedures were performed on live animals.

### Sampling area

Aguirre et al. [36] examined body shape variation in the Santa Rosa River (31–613 m) and from select museum samples from the Guayas and neighboring drainages. To these samples, we add samples collected in July 2014 from seven sites along an elevational gradient spanning from 50–1260 m in the Esmeraldas River drainage in northwestern Ecuador and five sites spanning from 69–1095 m in the Jubones River drainage in southwestern Ecuador (Fig 1). The Esmeraldas River was sampled because it is the drainage system from which *R*. *minor* was described and is known to harbor *Rhoadsia* throughout the drainage until at least 1260m above sea level. It is located in northwestern Ecuador between the Santiago-Cayapas and Guayas drainages and drains an area of approximately 21,418 km^2^ [48] from the western slope of the Andes to the Pacific Ocean in a southeast to northwest direction (Fig 1). Just south of it is the Guayas Drainage, which is the largest drainage in Western Ecuador draining an area of approximately 32,674 km^2^, and runs in a north to south direction funneled between the coastal mountain range (Cordillera Chongón-Colonche) and the Andes Mountains into the Gulf of Guayaquil [48, 49]. The Jubones River was sampled because of its geographic distance from the Esmeraldas drainage, being located close to the other extreme of the range of the genus *Rhoadsia* in southwestern Ecuador, and because of the presence and accessibility of *Rhoadsia* over a broad elevational range in this river. It is much smaller draining an area approximately 4,326 km^2^ [49] and further south than the Guayas, from which it is separated by many other small rivers running east to west between the Andes and the Pacific Ocean. The Jubones River also runs east to west along a relatively steep gradient into the Pacific. Immediately south of it is the Santa Rosa River drainage, which also drains a relatively small area approximately 756 km^2^ [36], and runs along a relatively steep gradient from east to west between the Andes Mountains and the Pacific Ocean (Fig 1). All of these drainages are isolated, impeding the migration of fishes between rivers, with the exception of flooding during severe rainy seasons that may allow some migration between rivers in southwestern Ecuador.

Samples were collected from seven sites across an altitudinal gradient in the Esmeraldas River drainage. These sites were selected to include the broadest altitudinal range of sites that were accessible and for which specimens of *Rhoadsia* were collected. There were three low elevation sites along the main branch of the river sampled at 50 m, 174 m, and 282 m above sea level respectively (Table 1). Samples were collected from two high altitude sites on each of two branches of the river above where it branched into the Río Toachi (southern branch) and Río Blanco (northern branch). The upstream sites were located at 668 m and 1095 m on the southern branch and 810 m and 1260 m on the northern branch. The site at 1260 m on the northern branch is Mindo (E6), which is the type locality for *R*. *minor* [35]. In the Jubones drainage, five sites were sampled along an altitudinal gradient at 69 m, 136 m, 251 m, 909 m, and 1095 m (Table 1, Fig 1). Although several attempts were made to collect between the 251 m and 909 m sites, much of the river in this area was not accessible or did not harbor *Rhoadsia*. The same was true for sites above 1095 m. Five sites were also sampled in the Santa Rosa River at 31 m, 86 m, 189 m, 382 m, and 613 m. Unfortunately, the branch of the river sampled was inaccessible above the 613 m site (see Aguirre et al. [36] for further details).

**Table 1:** Sampling sites and specimens collected. GM is the number of specimens in the geometric morphometric analysis of body shape, COI and S7i2 are the number of specimens sequenced for the mitochondrial COI gene and S7i2 specimens, respectively.

Site Code	Site Name	Elevation (m)	Latitude	Longitude (W)	GM	COI	S7i2
Esmeraldas							
E1	Afuera	50	0°35'02.9”N	79°36'38.9”	8	6	5
E3	Río Bravo	174	0°02'19.5”S	79°20'38.7”	10	10	7
E4	Valle Hermoso	282	0°04'44.1”S	79°17'05.6”	29	8	8
E5	Río Blanco	810	0°00'23.0”S	78°54'10.5”	8	8	0
E6	Mindo	1260	0°03'28.9”S	78°46'26.3”	56	13	8
E7	Meme Chico	668	0°13'26.1”S	79°04'19.8”	28	8	6
E8	Río Transito	1093	0°18'41.0”S	78°54'16.1”	21	8	8
Jubones							
J1	Huizho	69	3°19'40.0”S		27	9	7
J2	Casacay	136	3°19'48.2”S	79°42'40.9”	31	0	0
J3	Río Mollopongo	251	3°18'55.4”S	79°39'29.7”	33	6	8
J4	Río Minas	909	3°20'47.5”S	79°22'53.5”	20	8	0
J5	Río Mondur	1095	3°18'50.7”S	79°16'48.3”	25	10	8
Santa Rosa							
SR1	Santa Rosa1	31	3°30'06.0”S	29°57’25.0”	54	0	0
SR2	Santa Rosa2	86	3°33'31.4”S	79°56’45.5”	24	16	8
SR3	Santa Rosa3	182	3°34'53.0”S	79°54’44.8”	40	0	0
SR4	Santa Rosa4	382	3°35'23.9”S	79°50’33.1”	20	0	0
SR5	Santa Rosa5	613	3°35'09.3”S	79°48’32.6”	1	0	0
Guayas/Other							
GO1	Jauneche	50*	1°14’17.6”S	79°40’21.3”	15	15	7
GO2	Río Palenque	100*	0°34’26.3”S	79°21’43.5”	24	16	8
GO3	Balao Chico	50*	2°45’14.7”S	79°44’31.3”**	10	0	0
GO4	Chongón	50*	2°13’48.9”S	80°04’41.0”**	8	0	0
GO5	Churute	50*	2°17’57.3”S	79°38’19.2”	10	0	0
GO6	Salapi Chico	100*	0°39'38.1”S	79°32’09.8”**	5	0	0
Total=					507	141	88

*Approximate elevation.

**Approximate Latitude and Longitude.

### Field methods

Sampling was conducted using a Smith-Root Model LR24 electrofishing backpack (in combination with seines and dip nets) and settings were adjusted to maximize catch efficiency based on variation in local environmental conditions. Generally, electrofishing outputs ranged between 200–950 V with 25–30 Hz, with a standard pulse type and approximately 12–30% duty cycle. Electrofishing continued until 50 specimens were collected or 100 m of stream were sampled, whichever came first. The locations varied greatly in the abundance of *Rhoadsia* and 50 specimens could not be collected at all sites. After being euthanized, specimens were individually tagged and the right pectoral fin (and the caudal fin for small fish) were collected and preserved in 1.5 ml microcentrifuge tubes with 95% ETOH and stored at -80°C. The specimens were fixed in 10% formaldehyde for at least 24 hr, at which point samples were rinsed thoroughly in water and moved to 70% ETOH for long term storage. Preserved specimens and tissue samples were then transported back to DePaul University for morphometric and genetic analysis. Catalog numbers for the specimens deposited in museums are listed in the supplemental files (S1 Appendix). Data on water temperature, specific conductivity, oxygen concentration, pH, and water velocity were collected at each site (S1 Table). The data listed for the Santa Rosa River are based on measures taken in July 2013 and are averages of pool and riffle mesohabitats [36].

### Morphological analysis

Geometric morphometric methods were used to quantify body shape variation and generate visual depictions of body shape differences [42]. Specimens were straightened (if necessary) using insect pins and photographed on their left side with a 10.3 megapixel Nikon Coolpix P100 digital camera. Two dimensional coordinates were collected for 14 landmarks digitized on each specimen using TPSDIG, version 2.17 [50]. The landmarks were those used in Aguirre et al. [36], with the exception of two landmarks that were removed because of issues with the consistency of their placement across samples (S1 Fig). Although sexual dimorphism exists in this species, Aguirre et al. [36] found that it becomes pronounced in large males; small and intermediate sized males are similar in body shape to females. Therefore, sexual dimorphism was not taken into account in this study. The landmark data were aligned through Procrustes superimposition as implemented in Relative warps, version 1.53 [51] to eliminate variation related to rotation, translation, and size. Fins and eyes were drawn on the body shape outlines to facilitate visual interpretation of body shape variation. However, they are approximate and drawn only to provide anatomical references for the body shape landmarks.

To examine body shape divergence between the described species *R*. *altipinna* and *R*. *minor*, samples from the low elevation Guayas/Other drainages traditionally recognized as *R*. *altipinna* and specimens from the type locality of *R*. *minor* in Mindo, at 1260 m in the Upper Esmeraldas drainage were analyzed. Consensus configurations were created for each in Relative warps and the divergence in shape between the two species was modeled in Thin-plate spline version 1.20 [52] by depicting the landmark displacements necessary to transform the consensus configuration of *R*. *altipinna* into that of *R*. *minor*.

MANCOVA was conducted in tpsRegr 1.40 [53] to examine the contributions of allometry (shape variation associated with differences in body size—centroid size log_10_ transformed), drainage (Esmeraldas, Guayas/Other, Jubones, and Santa Rosa), and elevation (log_10_ meters above sea level) to the variation of body shape among all *Rhoadsia* samples. Centroid size was used because it is the most common measure of body size used in geometric morphometric analysis of fish body shape variation but using log_10_ standard length yielded very similar results because it was highly correlated with centroid size (r = 0.998). The significance of each factor in the MANCOVA was evaluated by running the complete model with and without the factor of interest and testing the significance of the difference in the residual sums of squares matrices. Wilks’ partial η^2^ was calculated following Langerhans and Dewitt [54] and Aguirre et al. [36] to estimate the relative contribution of these factors to body shape variation. Wilks’ partial η^2^ is a measure of the strength of the effect of a particular factor with larger numbers implying greater influence of a particular factor.

Canonical variates analysis (CVA) as implemented in MorphoJ 1.06d [55] was used to examine the major patterns of body shape divergence among samples. CVA is a standard multivariate method used to find the shape features that best distinguish among multiple predefined groups of specimens. Because allometry had a strong influence on body shape variation (see Results) and there was heterogeneity in size among samples (S2 Fig), the body shape data were size-corrected by conducting a pooled within-group regression of the body shape data on log_10_ centroid size. Site samples were used as the groups for this size-correction step and the residuals of the regression were used to conduct the CVA. Site samples were also used as the classifier variable for the CVA. The CVA was conducted on the individual specimen data but sample means computed from the individual scores on CV 1 and CV 2 were plotted in place of individual scores to reduce clutter. Landmark configurations depicting body shape divergence along CV 1 and CV 2 were included in the plot to provide a visual depiction of the body shape differences associated with the distribution of samples in the plot. To ensure that very small specimens present in some samples were not confounding the analysis, we repeated the same CVA on size-corrected data as described above but excluded all specimens under 40 mm SL. The analysis conducted on specimens >40 mm SL yielded the same general pattern as the analysis on all specimens so we retained all specimens in the analysis presented. We also conducted a principal components analysis (PCA) in MorphoJ, which yielded generally similar patterns of segregation of samples by elevation and drainage as obtained in the CVA (see Results) so we only present the results of the CVA.

To further examine how body shape varied with elevation in the Esmeraldas, Jubones and Santa Rosa rivers, for which samples were collected along elevational gradients, body shape was regressed on elevation separately for each river with centroid size (log_10_ transformed) included as a covariate in tpsRegr 1.40. Predicted body shapes at the lowest and highest elevation for each river were plotted with landmark displacements exaggerated by 3X to facilitate visualization. Wilks’ partial η^2^ values were computed as described above to give a measure of the impact on variation in elevation on body shape variation.

Finally, because body shape divergence among samples was largely associated with differences in body depth (see Results), the fineness ratio (FR) was also computed to facilitate comparisons with previous published data. FR is a simple measure typically used to describe body shape divergence between *R*. *altipinna* and *R*. *minor* [34, 38], with larger numbers indicating more streamlined bodies. It is calculated as the ratio of standard length (SL—measured from the tip of the snout to the end of the caudal peduncle) to body depth (BD—measured from the origin of the dorsal fin down to the ventral edge of the abdomen along a line perpendicular to SL). SL and BD were measured from the left side of the body of the digital images using tpsDig 2.17. *Rhoadsia altipinna* has typically been described as having FR of 2.25–2.5 while *R*. *minor* has ratios of 2.8–3.0 [38]. Because body shape is known to change significantly with size in this genus [36], we applied an allometric size correction following Reist [56]. Briefly, SL and BD were log_10_ transformed and predicted body depth at the grand mean standard length of all specimens in the study (56.8861 mm) was computed for each specimen using the equation: log _adj_BD = log BD_i_− β (log SL_i_−log SL_Gmn_), where log _adj_BD is the size adjusted specimen body depth, log BD_i_ is the body depth of the i^th^ specimen, β is the pooled within-groups slope computed as the weighted average of the slopes of the individual regressions of log BD against log SL for each of the 23 samples in the study (β = 1.058457), log SL_i_ is log_10_ of the standard length of the i^th^ specimen, and log SL_Gmn_ is the log_10_ of the grand mean standard length of all specimens included in the study. Fineness ratios were then computed for all specimens from the ratios of the size-adjusted BD by the SL_Gmn_ and averaged by sample.

### Genetic analysis

Fin clips were preserved in 95% ethanol in the field and stored at -80°C until DNA extraction. In the lab, DNA was extracted from fin clips using a standard phenol:chloroform:isoamyl alcohol protocol following Aguirre et al. [36]. A fragment of the mitochondrial cytochrome oxidase I gene was amplified following Aguirre et al. [36]. The primers used for the second intron of the S7 ribosomal protein gene (S7i2) were: S7RPEX2F: 5’-AGCGCCAAAATAGTGAAGCC-3’ and S7RPEX3R: 5’-GCCTTCAGGTCAGAGTTCAT-3’ [57]. The PCR conditions followed those described for the COI gene in Aguirre et al. [36] except for the annealing temperature, which was 60°C. PCR products were purified using ExoSAP-IT^®^ (USB Corporation). Forward and reverse strands were sequenced on an Applied Biosystems 3730 DNA Analyzer at the University of Arizona Genetics Core. Sequences were aligned with ClustalW as implemented in BioEdit 7.2.3 [58] and alignments were manually inspected for errors with Chromas Lite (Technelysium, Pty Ltd., South Brisbane, Queensland, Australia; http://www.technelysium.com.au/chromas_lite.html). After editing, the COI fragment was 606 bp long while the S7i2 fragment was 316 bp long.

A maximum parsimony median joining network depicting relationships among alleles was constructed using Network 5.0.0.0 (Fluxus Technology Ltd., Suffolk, England; http://www.fluxus-engineering.com/sharenet.htm). Transversions were weighted two times over transitions and the default ConnectionCost distance calculation method and the post-processing MP calculation were employed. Haplotype and allele richness (H & A, the number of haplotypes and alleles per population) were calculated for each sample, as were haplotype diversity (H_d_) calculated as N(1-Σp_i_^2^)*(N-1)^-1^, where p_i_ is the frequency of the i^th^ allele and N is the number of individuals in the sample [59], the effective number of alleles (A_e_) calculated as 1/(Σp_i_^2^), where p_i_ is the frequency of the i^th^ allele [60], and the number and percentage of private haplotypes and alleles (H_p_ and A_p_, haplotypes and alleles found exclusively in the i^th^ sample). Arlequin 3.5.1.2 [61] was used to calculate pairwise F_ST_ values between populations and conduct an AMOVA to examine the components of genetic variation associated with divergence among river drainages, divergence among populations within drainages, and divergence among individuals within populations.

Making decisions on species delimitation can be challenging in some cases when data are limited and provide unclear results [62, 63]. Dealing with allopatric populations can be particularly difficult because this is a situation in which the most common species definition applied to vertebrates, the biological species concept, performs particularly poorly. As a first step, we evaluate three general scenarios in our examination of the genetic data using the criterion of reciprocal monophyly from the phylogenetic species concept (PSC). First, *Rhoadsia altipinna* and *R*. *minor* may be two highly divergent species genetically in which case we would expect reciprocally monophyletic clades for populations corresponding to *R*. *altipinna* and *R*. *minor* and large distances (>1%) among the most similar alleles between clades. Since there are questions about the distribution of *R*. *minor*, we evaluated this criterion using three possible ranges for *R*. *minor*: the entire Esmeraldas drainage, the high elevation localities in the Esmeraldas (>600 m), and the type locality for *R*. *minor* at the Mindo locality. Second, *Rhoadsia* may be a single, genetically panmictic but morphologically variable species that occurs throughout western Ecuador, in which case we would expect no monophyletic clades identifiable as *R*. *altipinna* or *R*. *minor*, and no or little significant genetic variation associated with drainages or elevation. Finally, an intermediate scenario is possible, in which populations assigned to *R*. *altipinna* and *R*. *minor* exhibit significant genetic divergence, but of lower magnitude than typically seen among sister species, and thus appear to be somewhere along the speciation continuum [9, 64]. In this case, we would expect significant population genetic structure among populations corresponding to the species but no reciprocal monophyly or deep structure among alleles.

## Results

### Body shape variation

**Fig 3:**
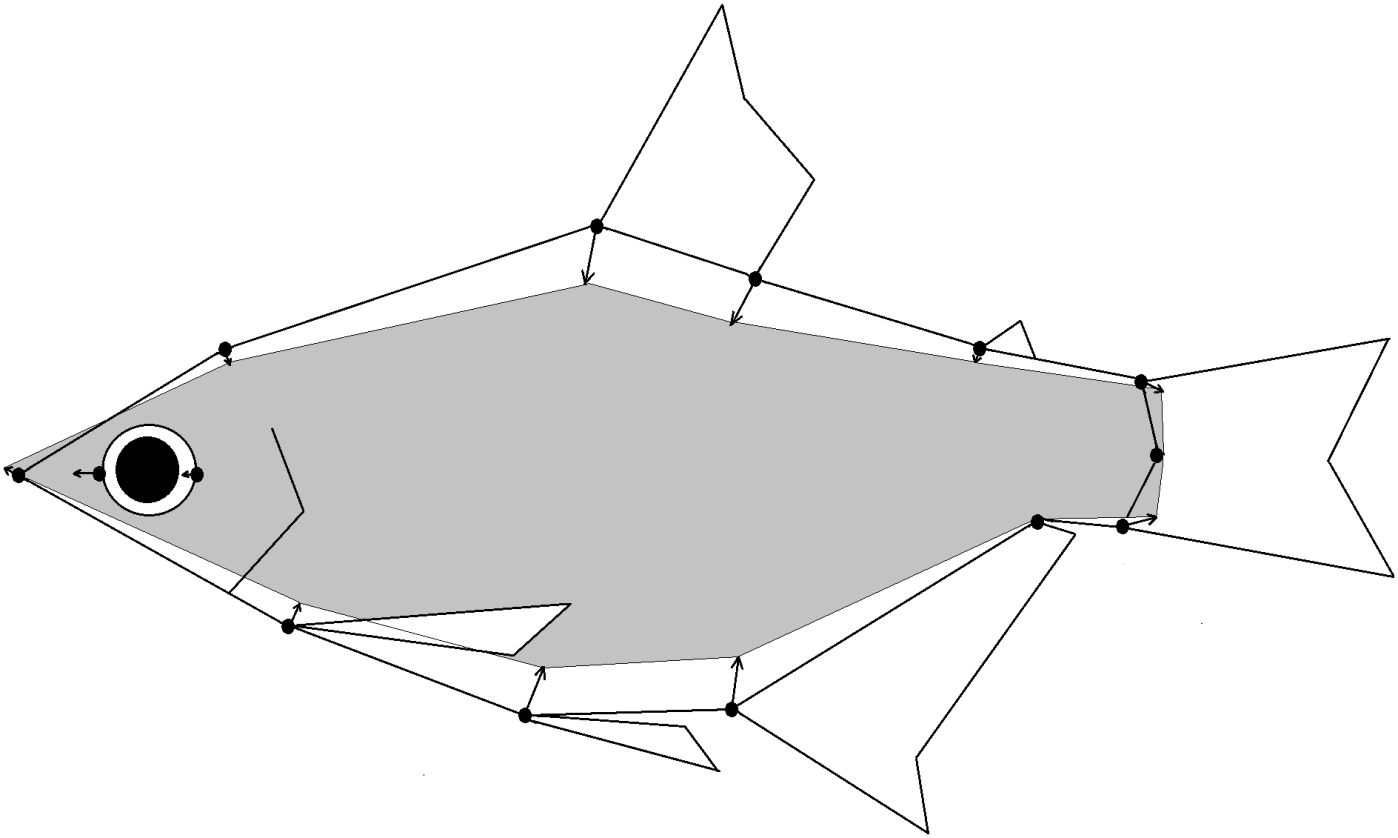
Comparison of the outline of the consensus configuration for the *R*. *altipinna* Guayas/Other samples showing the shape difference relative to the consensus configuration for the *R*. *minor* Mindo sample (type locality, E6), which is shaded in gray. Analysis conducted using Thin-plate spline version 1.2. Fins and eyes are approximate and included only for reference to facilitate interpretation of body shape variation.

*Rhoadsia altipinna* and *R*. *minor* have traditionally been described as differing primarily in body depth. Our geometric morphometric analysis of body shape divergence between the samples illustrating the classic representatives for these species, the consensus configurations of the Guayas/Other low elevation *R*. *altipinna* and the high elevation *R*. *minor* specimens from the type locality, strongly confirms this (Fig 3). The most significant landmark displacements between the consensus configurations involved the landmarks associated with the dorsal fin moving ventrally, and the landmarks at the insertion of pelvic fin and origin of the anal fin moving dorsally in *R*. *minor* relative to *R*. *altipinna*. Combined with the landmarks at the origin of the adipose fin and the posterior margin of the cranium moving ventrally, and the landmark at the insertion of the pectoral fin moving dorsally, these displacements indicated a strong dorsal-ventral compression in *R*. *minor* relative to *R*. *altipinna* that was most pronounced in the mid region of the body. Other landmarks that exhibited conspicuous displacements included those at the dorsal and ventral margins of the caudal peduncle moving posteriorly, and the landmarks at the margins of the orbital and tip of the snout moving anteriorly in *R*. *minor* relative to *R*. *altipinna*, resulting in an anterior-posterior elongation of *R*. *minor* relative to *R*. *altipinna*.

Examining body shape variation across all samples, allometry, drainage and elevation were all associated with significant and substantial components of variation in body shape (Table 2). Based on the Wilks’s Partial η^2^ measures, allometry had the most substantial influence on body shape variation followed by drainage and elevation, which had effects of similar magnitude. Given the influence of allometry on body shape variation, subsequent analyses examining shape divergence among samples were based on size-adjusted data.

**Table 2:** MANCOVA of body shape variation of *Rhoadsia* attributable to Allometry, Drainage, and Elevation. Wilks’s Partial η^2^ is a measure of the importance of a particular factor with larger numbers implying greater importance. All factors were significant at P<0.0001.

	Wilks’s λ	Fs	DF1	DF2	Wilks’s Partial η^2^
Allometry	0.143	119.4	24	478	0.857
Drainage	0.138	18.6	72	1429	0.483
Elevation	0.539	17	24	478	0.461

**Fig 4:**
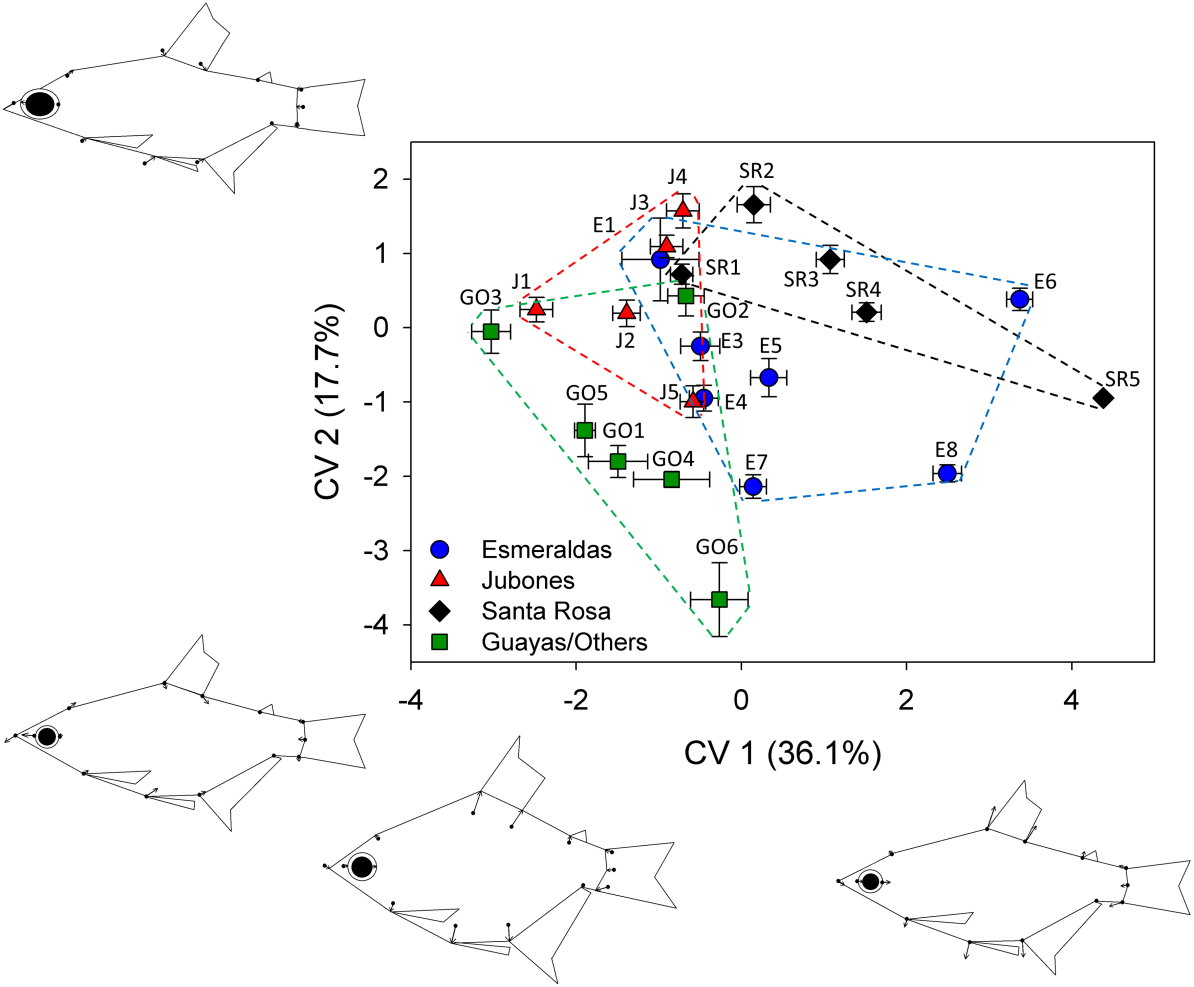
Canonical variate analysis (CVA) of size-adjusted body shape data. Percentages are the percentage of variation among groups accounted for by each axis. Points are sample means and error bars are standard errors of the mean. Landmark configurations depict body shape divergence along the CV axes. Figures on the bottom left and right represent predicted shape divergence along CV 1. Figure on the top left represents shape divergence along CV 2 with darker outline representing more negative values and lighter outline indicating more positive values. Shape deformation exaggerated 10X. Fins and eyes are approximate and included only for reference to facilitate interpretation of body shape variation.

The CVA on the size-corrected shape data indicated substantial divergence among samples by both elevation and drainage (Fig 4). Body shape variation associated with divergence along CV1 accounted for 36.1% of the body shape variation among groups, and was largely attributable to differences in body depth consistent with that described above for the classical *R*. *altipinna*-*R*. *minor* divergence. Negative values along this axis indicated deeper bodies while positive values indicated more streamlined bodies. Other slight differences included an expansion of the base of the anal fin and a decrease in the base of the dorsal fin with more positive values along CV1. The Guayas/Other samples (GO), which were collected at low elevations, tended to have more negative values along CV1, consistent with their deeper bodies. Samples collected at lower elevations in the Jubones, Santa Rosa and Esmeraldas drainages tended to also have more negative values along CV1, with sample means shifting to more positive values as elevation increased. This pattern was most pronounced in the Santa Rosa and Esmeraldas samples, in which the highest elevation Santa Rosa sample and the two highest elevation Esmeraldas samples exhibited the most extreme positive values on this axis. CV2 accounted for 17.7% of the body shape variation among groups and was also somewhat associated with variation in body depth with landmarks 3 and 4 marking the base of the dorsal fin moving down and posteriorly and landmark 10 marking the anterior base of the anal fin and landmark 11 marking the insertion of the pelvic fin moving up and posteriorly with more positive values of CV2. The snout also increased in length, the eye increased substantially in diameter and the caudal peduncle decreased in length with more positive values on CV2. There was no obvious pattern to segregation among samples by elevation or drainage along CV2.

**Fig 5:**
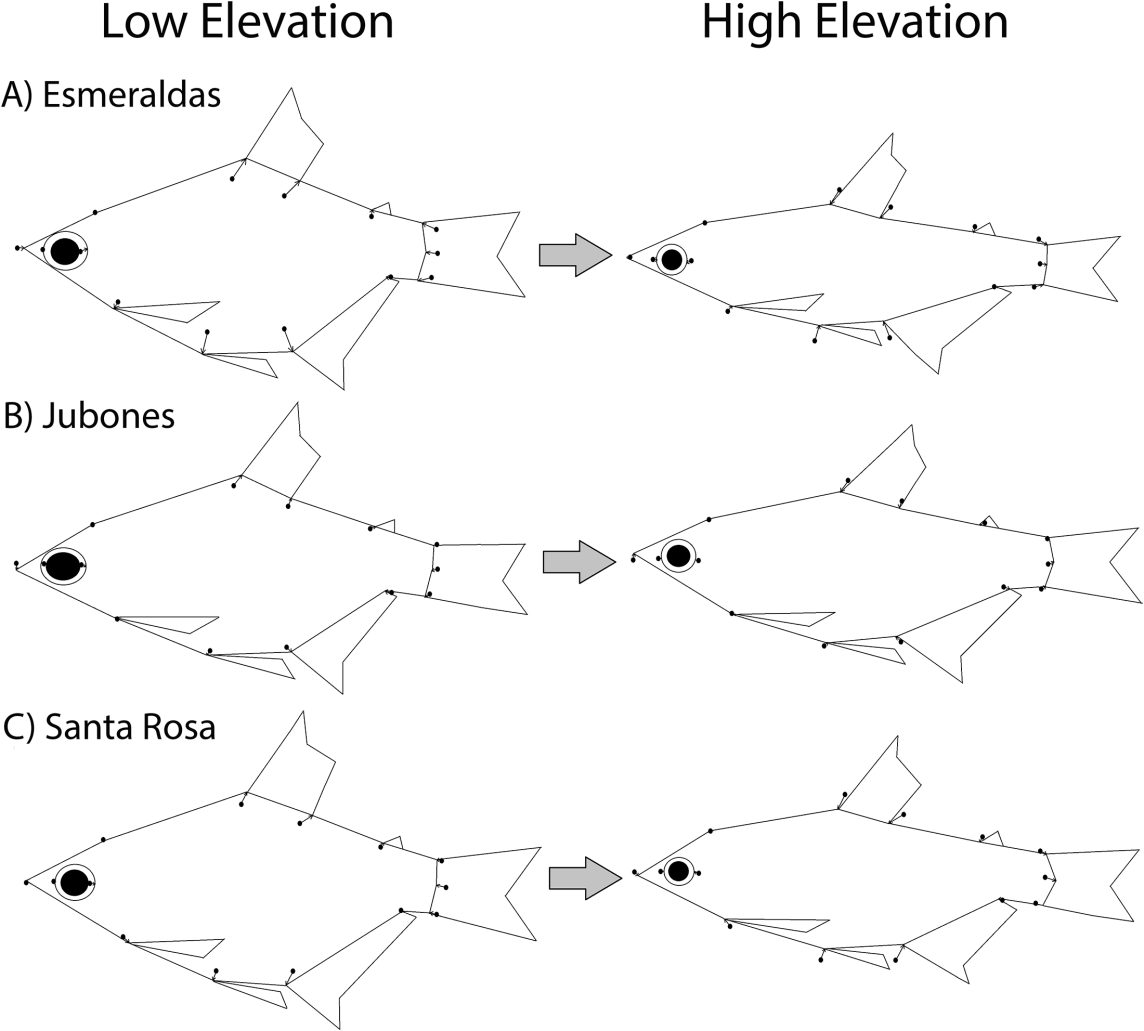
Predicted body shape of specimens at the lowest (left) and highest (right) elevations in the (A) Esmeraldas, (B) Jubones, (C) and Santa Rosa rivers from regression analyses conducted separately on samples from each river. Vectors depict the landmark displacements from the average for each river. Fins and eyes are approximate and included only for reference to facilitate interpretation of body shape variation. Body shape divergence exaggerated 3X.

Regression of body shape on elevation in the three rivers for which sampling was conducted along elevational gradients confirmed the occurrence of parallel streamlining of the body with elevation in all of them (Fig 5). As seen for the general MANCOVA, centroid size still had the strongest effect on body shape variation in all rivers. However, increase in elevation was associated with a large and statistically significant increase in streamlined bodies across all rivers (Wilks’ partial η^2^ = 0.719 in the Santa Rosa River, 0.702 in the Esmeraldas River, and 0.617 in the Jubones River, P<0.0001 in all rivers). Although the change in body depth was most apparent, there were other small differences in body shape with elevation. For example, there was a slight forward movement of the dorsal fin and decrease in the diameter of the eye with elevation in all drainages. In the Esmeraldas and to a lesser extent in the Jubones drainage, there was an expansion in the ventral region of the abdomen at lower elevations as evidenced by displacements at landmarks 10 and 11. In the Santa Rosa drainage and to a lesser extent in the Esmeraldas drainage, there were declines in the length of the base of the anal fin and dorsal fin with elevation.

**Fig 6:**
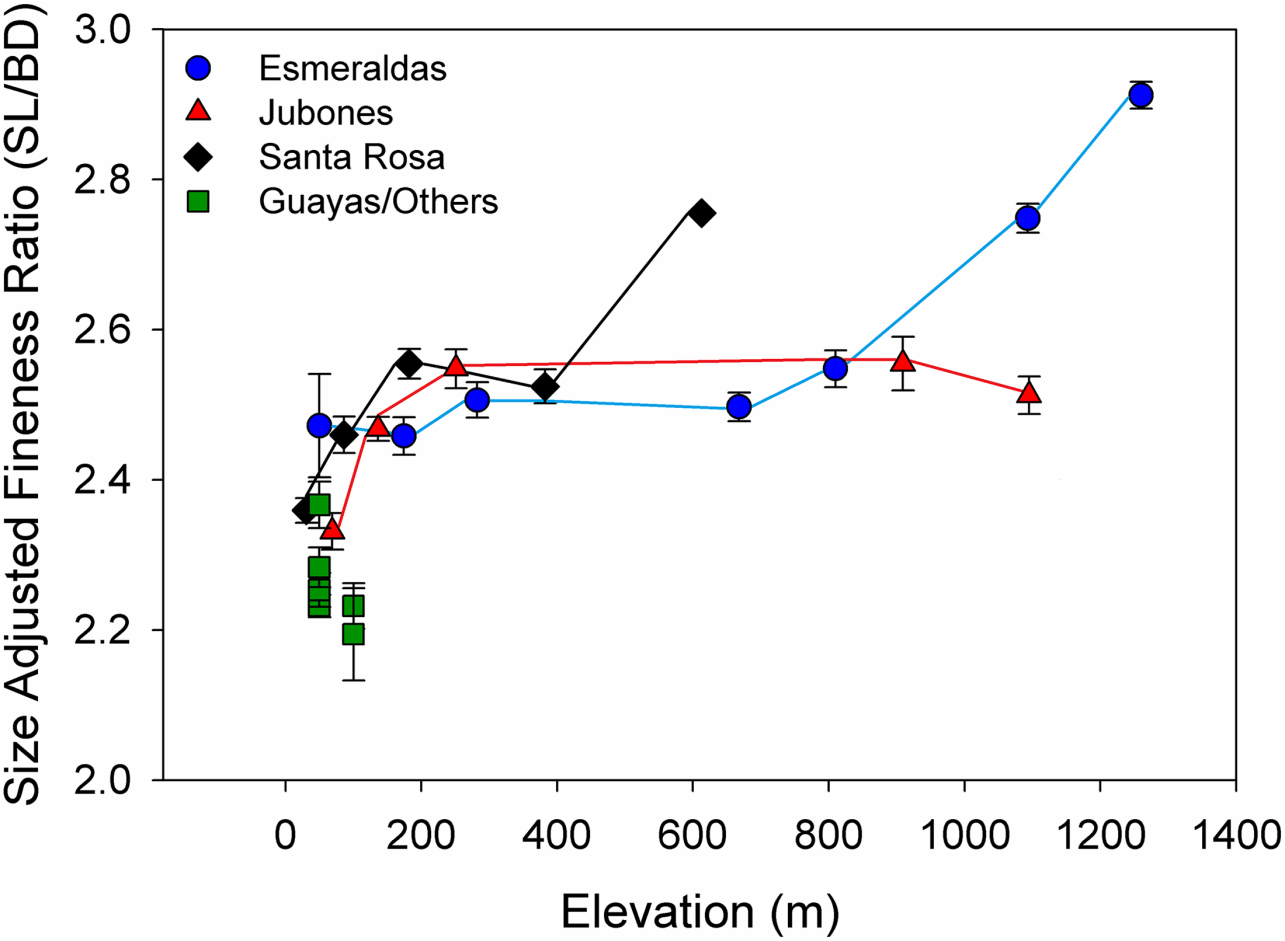
Variation in mean size-adjusted fineness ratio with elevation with samples coded by drainage. Fineness ratio is a measure of body elongation with larger numbers indicating more streamlined bodies. Error bars are standard errors of the mean.

The change in body depth was also apparent when examining variation in FR (SL/BD), a simpler metric that has been more traditionally used to examine differences among *Rhoadsia* spp. (Fig 6). Size-adjusted mean FRs for the low elevation Guayas/Other samples varied between approximately 2.2 and 2.4, in accordance with expectations for *R*. *altipinna*. FR increased with elevation in the drainages for which samples were collected along elevational gradients but the pattern of change differed among drainages, consistent with the differences in body shape among drainages documented in the CVA. The lowest elevation sites in both the Santa Rosa and Jubones drainages had mean FR that were similar to the higher values for the Guayas/Other drainages, but these quickly increased at the next upstream sites, remaining fairly stable between the 251 and 1095 m sites in the Jubones River, but increasing substantially between the 382 and 613 m sites in the Santa Rosa River, with the latter being very close to the cutoff of 2.8 for *R*. *minor*. It is worth noting that the 613 m Santa Rosa site is based on a single specimen so it should be interpreted with caution. The mean FR also increased with elevation in the Esmeraldas drainage although the pattern is quite different. The lowest elevation site in the Esmeraldas collected at 50 m already had an elevated mean size-adjusted FR relative to the Guayas/Other samples and the lowest elevation Santa Rosa and Jubones samples. Mean FR then remained relatively stable through the 668m site, increasing slightly at 810 m, before increasing much more dramatically at the 1093 and 1260 m sites. It was only the two highest elevation sites that had mean FR close to (the E8 1093 m site) or within (the E6 1260 m site) the 2.8–3.0 range typically used to distinguish *R*. *minor*.

### Genetic variation

**Fig 7:**
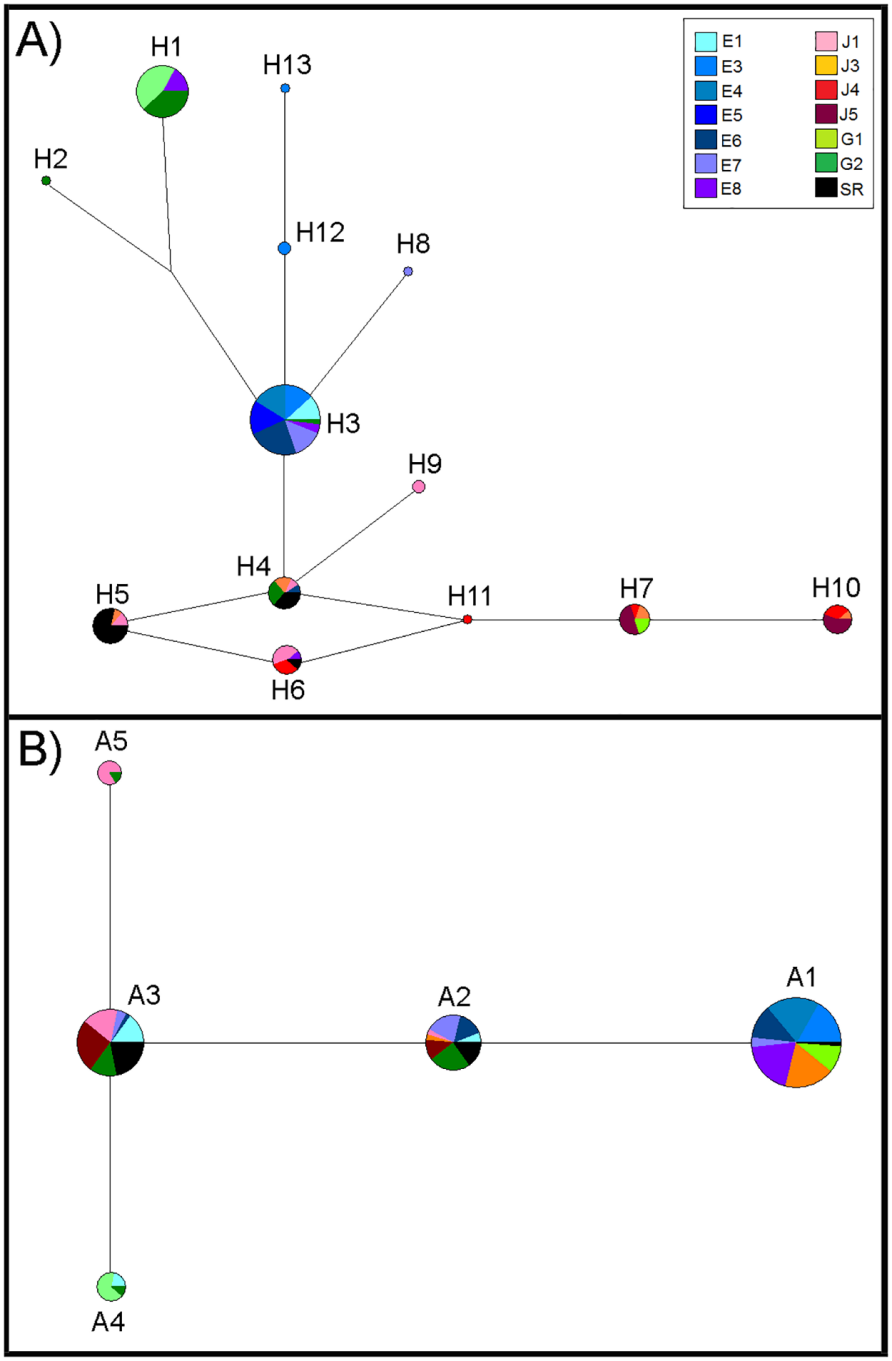
Genetic networks constructed using parsimony analysis in Network 5.0.0.0. Sizes of the circles are proportional to the number of specimens included. A) Network for COI haplotypes. B) Network for S7 intron alleles.

Genetic diversity was relatively low for both genes (Table 3, S2 and S3 Tables). There were 13 unique haplotypes recovered for the COI gene resulting from 11 mutations among the 141 specimens sequenced. Ten of these mutations were transitions and one was a transversion. Of the transitions, nine were in third codon positions while one was in a first codon position. None of the mutations resulted in amino acid changes. The maximum sequence difference between any pair of haplotypes was six nucleotides or 0.99% and most haplotypes were separated from their nearest neighbor by one mutation. None were separated by more than two mutations (Fig 7A). For the S7i2 gene, there were only five alleles resulting from four mutations for the 88 specimens sequenced. Of these mutations, two were transitions and two were transversions. The maximum sequence difference between any pair of haplotypes was three nucleotides or 0.95% and all alleles were separated from their nearest neighbor by one mutation (Fig 7B).

**Table 3:** Genetic diversity of COI and S7i2 genes. N is the number of specimen sequenced per sample and per gene, H is the number of COI haplotypes, H_d_ is haplotype diversity, H_p_ is the number (and percentage) of private haplotypes, A is the number of alleles, A_e_ is the effective number of alleles, and A_p_ is the number (and percentage) of private alleles.

COI					S7i2			
Site	N	H	H_d_	H_p_ (%)	N	A	A_e_	A_p_ (%)
E01	6	1	0.00	0	5	3	2.3	0
E03	10	3	0.51	2 (66.6)	7	1	1.0	0
E04	8	1	0.00	0	8	1	1.0	0
E05	8	1	0.00	0	-	-	-	-
E06	13	2	0.15	0	8	3	2.0	0
E07	8	2	0.25	1 (50.0)	6	3	2.3	0
E08	8	3	0.61	0	8	1	1.0	0
J01	9	4	0.78	1 (25.0)	7	3	2.2	0
J03	6	4	0.87	0	8	2	1.1	0
J04	8	4	0.79	1 (25.0)	-	-	-	-
J05	10	2	0.56	0	8	2	1.6	0
G01	15	2	0.25	0	7	2	2.0	0
G02	16	4	0.52	1 (25.0)	8	4	2.5	0
SR	16	3	0.49	0	8	3	2.0	0
Total	141				88			

Beyond the relatively low genetic diversity and small distances among alleles, we failed to recover monophyletic clades corresponding to samples falling within the described species *R*. *altipinna* and *R*. *minor* for either gene (Fig 7, S2 and S3 Tables). Specifically, there was no monophyletic clade corresponding to *R*. *minor* for the Esmeraldas drainage, the high elevation Esmeraldas samples (samples collected at 668 m or higher), or even for the type locality of *R*. *minor* at Mindo (Sample E6). For example, for the COI gene, haplotype H4 was shared by fish from all four drainage systems and the Esmeraldas specimen of this haplotype was collected at the type locality for *R*. *minor*. Haplotype H3, which was the most common haplotype by far in the Esmeraldas drainage, also occurred in a specimen from the Guayas drainage (site G2). Haplotype H1, which was the most common haplotype in the Guayas drainage also occurred in the Upper Esmeraldas drainage (E8). Haplotype H6 was shared by fish in the Esmeraldas (E8), Jubones and Santa Rosa drainages. Moreover, the most common haplotypes in the Guayas (H1) and Esmeraldas (H3) drainages differed by only two mutations, while the most common haplotype in the Santa Rosa River (H5) differed by two mutations from H3 and by four mutations from H1. There was not a dominant haplotype in the Jubones drainage and all haplotypes present in Jubones specimens were within four mutations from the common Esmeraldas H3 haplotype. The sharing of alleles among drainages was even more common for the S7i2 gene with the three major alleles (A1-A3) occurring in specimens of all four drainages.

Despite the lack of monophyly, we did find significant population genetic structure among the samples. AMOVA indicated that there was a significant component of genetic variation that segregated among drainages for the COI gene but not for the S7i2 gene (Table 4). For the COI gene, 58.2% of the genetic variation segregated among drainages indicating substantial divergence among the drainages sampled. This was followed by 30.4% of the variation segregating among individuals within sites and 11.4% of genetic variation segregating among sites within drainages. The genetic divergence among drainages was apparent in that the most frequent haplotypes were different among drainages. As indicated above, H1 was the most common haplotype in the Guayas drainage, H3 was the most common drainage in the Esmeraldas drainage, and H5 was the most common haplotype in the Santa Rosa drainage. However, none of these were exclusive to one drainage. Pairwise F_ST_ values also seemed associated with the geographic distances among sites (S4 Table). For the S7i2 gene, the greatest proportion of genetic variation segregated among sites within drainages (53.8%), followed by variation segregating among individuals within sites (41.9%). Variation attributable to divergence among drainages was small (4.3%) and not statistically significant. The divergence in allele frequencies between most sites was significant but seemed idiosyncratic and was not obviously associated with differences in elevation among the sites (S3 and S4 Tables).

**Table 4:** AMOVA of COI (top) and S7 (bottom) sequence data.

COI				
Source of variation	df	Sum of squares	Percentage of variation	P
Among drainages	3	84.5	58.2	<0.0001
Among sites within drainages	10	18.2	11.4	<0.0001
Within sites	127	52.3	30.4	<0.0001
Total	140	155.0		
S7				
Source of variation	df	Sum of squares	Percentage of variation	P
Among drainages	3	18.3	4.3	0.36
Among sites within drainages	8	38.7	53.8	<0.0001
Within sites	164	40.7	41.9	<0.0001
Total	175	97.7		

## Discussion

The existence of two closely related species of *Rhoadsia* reported from different elevations and differing substantially in an adaptively important trait like body depth, provides an opportunity to gain insight into the process of adaptation and speciation in Neotropical mountain streams. Unfortunately, considerable uncertainty exists concerning the distribution of phenotypic variation and the relationship between the two species of *Rhoadsia*. In this study, we found that the main character distinguishing the two described species, body depth, varies continuously among populations within drainages as a function of elevation, and that body shape overlaps among drainages, such that low elevation populations of *R*. *minor* in the Esmeraldas drainages have similar body depths to higher elevation *R*. *altipinna* in southern drainages. Although a common general trend of declining body depth with elevation is clear, the pattern and magnitude of body shape divergence differed among drainages and is most extreme in the Esmeraldas River drainage, from which *R*. *minor* was originally described, indicating significant heterogeneity among drainages in the factor(s) driving body shape divergence. Sequencing of mitochondrial and nuclear genes failed to produce monophyletic lineages corresponding to the two described species and the structure among alleles in both genes was shallow, suggesting relatively recent genetic divergence on a geological time scale. However, there was a large component of genetic variation that segregated among drainages for the COI gene, indicating significant genetic divergence associated with geographic isolation. The genetic and morphological data seem generally consistent with *Rhoadsia* spp. falling somewhere along the speciation continuum [9, 64], although where is not yet clear. Below we discuss the potential causes of body shape divergence documented, the implications of our results for species delimitation, and potential directions for future research.

### Causes of body shape change with elevation in *Rhoadsia*

Cases of naturally replicated divergent habitats can provide particularly important insights into evolution and speciation. There are now many documented cases of parallel phenotypic divergence in which species have repeatedly evolved similar characteristics after independently colonizing similar types of habitats [65–68]. Although each mountain stream in western Ecuador has elements to its history, structure, and ecology that are unique, they share many elements, including common patterns of continuously changing habitat characteristics along elevational gradients [18–20]. The repeated declines in body depth with elevation that *Rhoadsia* populations exhibit are consistent with previous reports of parallel phenotypic divergence in response to common patterns of environmental change. Since genetic drift would not likely produce consistent directionality of phenotypic divergence with elevation, this mechanism is unlikely to be driving the pattern observed. Whether the phenotypic changes are the result of phenotypic plasticity or adaptive evolution is unclear, and future common garden experiments or related approaches will have to tease this apart. However, it is worth noting that adaptive phenotypic plasticity may facilitate adaptive evolution in some cases [14, 69], and phenotypic differences that originate through phenotypic plasticity may sometimes be integrated into the genome through the process of genetic assimilation [14, 70–72]. Adaptive evolution and phenotypic plasticity are thus not necessarily exclusive alternatives for phenotypic diversification.

What is driving the parallel decline in body depth with elevation? Aguirre et al. [36] discuss several possible causes including differences in water velocity, predator abundance, and sexual selection. Water velocity and predation are particularly interesting possibilities because both of these factors have been shown to be associated with changes in body shape that are similar to those documented here and vary with elevation. Habitats with fast-flowing water tend to be more abundant at higher elevations where stream gradients are higher, while predatory fishes tend to be more abundant at lower elevations [19, 23]. Faster flowing water and low fish predation both select for more streamlined bodies while slower flowing water and high fish predation select for deeper bodies [37], consistent with the pattern seen in *Rhoadsia* populations. Better quantification of water flow variation and predator abundance along elevational gradients and experimental tests are necessary to clarify the potential roles of these factors.

Another factor that may play a role but was not mentioned in Aguirre et al. [36] is temperature. Water temperature can have a large influence on the development of ectotherms and is known to impact body shape and the axial skeleton in fishes. In Neotropical mountain streams, water temperature declines significantly with elevation [19], and this was the case for the rivers sampled along elevational gradients in this study. Water temperature was strongly negatively correlated with elevation with maximum and minimum water temperatures varying between 29.3 and 19.5°C (r = -0.981, elevation log_10_ transformed for all rivers) in the Esmeraldas drainage, 24.2 and 20.1°C (r = -0.750) in the Jubones drainage, and 24.4 and 19.0°C (r = -0.755) in the Santa Rosa drainage (S1 Table, S3 Fig). Interestingly, the greatest temperature range was recorded in the Esmeraldas River, where divergence in body shape with elevation was most extreme. Recent studies indicate that water temperature can impact fish body shape in a manner consistent with the pattern documented here. For example, Georgakopoulou et al. [73] found that European seabass *Dicentrarchus labrax* raised at 15°C had significantly more slender bodies than when raised at 20°C. More recently, Reyes [74] found that specimens of the characid *Astyanax mexicanus* grown at 20°C had significantly more streamlined bodies than fish grown at 23, 25, or 28°C. Why lower temperatures result in more streamlined bodies in fishes is unclear, but lower temperatures can also result in an increase in vertebral number (Jordan, 1891; McDowall, 2008), and increases in vertebral number are known to be associated with body elongation both at broad taxonomic ranks [75–77] and within species [77]. Thus, whatever the biochemical mechanism, temperature variation during development can produce phenotypic effects like those documented in this study for *Rhoadsia* and warrants further investigation. Many other factors vary with elevation in Andean mountain streams [19] and could be contributing to the pattern documented. It is beyond the scope of this study to address them all. This is an important direction for future research.

Another potential direction for future research is to survey more rivers along elevation gradients to examine the extent of variation among rivers in the pattern of *Rhoadsia* body depth change with elevation and the causes of this variation. Although the general trend of decline in body depth at higher elevations was shared across all rivers in this study, the pattern was different in each. *Rhoadsia* in the Esmeraldas drainage tended to exhibit more streamlined bodies per unit of elevation and the high elevation sites had the most streamlined bodies documented (Fig 6). The two highest sites sampled in the Jubones River were comparable in elevation to the highest sites sampled in the Esmeraldas. So why was the decline in body depth not greater at the high elevation sites in the Jubones? There are many differences between the Esmeraldas drainage and other drainages in southwestern Ecuador like the Jubones including 1) its size: the Esmeraldas drainage is the second largest drainage in western Ecuador, only behind the Guayas drainage, and approximately five times larger than the Jubones; 2) the gradient: elevation increases much more quickly in the Jubones because of the proximity of the Andes to the ocean in the region (Fig 1, S4 Fig); 3) precipitation: the Esmeraldas receives more precipitation than the drainages south of it; 4) species diversity: the Esmeraldas has substantially more fish species than the Jubones; etc., [28]. Greater sampling is needed to determine whether differences between rivers in the pattern of morphological change can be attributed to particular factors.

### Where do the divergent *Rhoadsia* populations fall along the speciation continuum?

Species are the fundamental units of biodiversity so delimiting them properly is crucial. However, speciation is not an instantaneous event. Populations in nature can fall anywhere along a “speciation continuum” from exhibiting continuous variation with no reproductive isolation to exhibiting adaptive divergence and irreversible reproductive isolation [64, 78]. Although DNA barcoding has proved to be an extremely useful tool for species identification and delimitation [43, 79, 80], making reliable decisions on species delimitation can be very challenging and often requires a comprehensive examination of many different types of data beyond those available here [62, 63, 81]. We outlined three general scenarios based on the genetic data as a first step towards evaluating whether *Rhoadsia* in western Ecuador should be considered one or two species. Our data conclusively eliminated the two simpler scenarios. *Rhoadsia altipinna* and *R*. *minor* are clearly not highly divergent, ancient species under a strict interpretation of the phylogenetic species concept given the lack on reciprocal monophyly and the shallow structure of the alleles for both genes. Nor is there a single genetically panmictic population of *Rhoadsia* occurring throughout western Ecuador, given the significant population genetic structure associated with drainages for the mitochondrial COI gene and the genetic distinctiveness of the Esmeraldas drainage samples. These were dominated by the H3 haplotype (frequency = 82% in the Esmeraldas drainage) that was only present in one individual from outside the Esmeraldas drainage, a specimen from the neighboring Guayas drainage. It is worth noting that we found no evidence of a high elevation and low elevation species pair. Thus genetically, we are left with the difficult, intermediate scenario: closely related allopatric populations exhibiting significant population genetic structure associated primarily with the geographic isolation of river drainages.

Until more data are available, we suggest that the two species of *Rhoadsia* continue to be recognized, with *R*. *minor* corresponding to populations in the Esmeraldas drainage (and possibly drainages to the north of the Esmeraldas) and *R*. *altipinna* corresponding to populations from the Guayas drainage south into northern Peru as currently recognized [27, 28]. The magnitude of the divergence in haplotype frequencies for COI indicates substantial genetic isolation between populations in the Esmeraldas vs. southern drainages. Since genetic isolation is one of the most important factors facilitating evolutionary divergence [1], populations in the Esmeraldas may differ at adaptively important genes from populations in southwestern Ecuador. The morphological distinctiveness of individuals collected at high elevations in the Esmeraldas drainage suggests that genes for body shape evolution may be among these, assuming that the difference in body shape has a genetic component (something that needs to be tested). Additionally, there are no data on the ecology or breeding habits of *Rhoadsia* populations, so we cannot rule out that the Esmeraldas and Guayas populations have evolved significant ecological or reproductive differences that might qualify them as distinct species under other species concepts [1]. There are many cases of populations that are closely related genetically but have evolved reproductive isolation or other differences that qualify them as distinct species [82–84].

Having found a clear pattern of parallel body shape divergence with elevation across drainages and a lack of strong genetic divergence or reciprocal monophyly between the two described species, we can make specific recommendations for future studies on the delimitation of the species. First, it is clear that genetic markers with higher rates of evolution should be employed and that larger samples should be surveyed. If the two species are valid, they are closely related genetically and methods more closely associated with population genetics will likely be appropriate. We specifically suggest using microsatellite markers for which primers have already been designed [39] and mitochondrial markers like cytochrome b that are known to have higher rates of evolution than COI. SNPs screened using genomic methods could also be employed. Given the close relatedness of the populations, use of nuclear markers is critical to avoid issues with the effects of introgression on mitochondrial markers. Second, the phylogenetic species concept or other species concepts that incorporate criteria more appropriate for dealing with closely related allopatric forms than the biological species concept should be employed. Third, other data on the ecology, reproductive habits, and/or the genetic basis of the morphological differences among populations should be collected to help inform the species delimitation decision. Since there was relatively strong divergence in the frequencies of haplotypes of the COI gene between the Esmeraldas samples and those in other drainages, molecular markers with higher rates of evolution may yield reciprocally monophyletic clades segregating by drainage. However, most recent reviews dealing with delimitation of closely related allopatric species emphasize the importance of evaluating multiple sources of evidence and not relying exclusively on a measure of genetic distance to define species [62, 63].

## Conclusions

The properties that *Rhoadsia* populations exhibit, including variation in an adaptively important trait that is generally important across fishes, the relatively low genetic distances among populations that differ substantially in phenotype, and the occurrence of replicated phenotypic gradients in independent river drainages, all make a case that *Rhoadsia* has the potential to provide significant insight into the process of adaptation and speciation in Neotropical mountain streams. However, there is much that remains to be learned. As we indicated above, the basis of the phenotypic divergence (genetically based or phenotypic plasticity) remains to be identified as do the factor(s) causing the replicated patterns of body shape divergence. Studies examining whether intrinsic or extrinsic mechanisms for reproductive isolation have evolved in geographically isolated *Rhoadsia* populations are greatly needed. Future genetic studies should include markers with higher rates of evolution like microsatellites or other mtDNA genes [60, 85]. Unfortunately, as is the case for most fishes in western Ecuador [86, 87], very little is known about its basic ecology and life history for this characid, and filling in the gaps in knowledge will be crucial for understanding the evolutionary processes at play.

With the continued development of Next-Gen sequencing methods, an interesting direction of future research is to examine the genetic basis of adaptation along elevational gradients. Are there genes of major effect for adapting along elevational gradients in Neotropical mountain streams? Are the same genes or different genes involved in adaptation to high elevations in different drainages? Can adaptive alleles be shared (recycled) among high elevation populations through gene flow with low elevation populations [88, 89]? Studies of this nature have provided great insight into the genetic basis of parallel evolution in other taxa and could be pursued in *Rhoadsia* [90–95].

## Supporting information

S1 Fig Landmarks and linear measures used in the study. Insect pins were used to mark some of the landmarks that were difficult to see from a lateral perspective. SL is standard length and BD is body depth. The specimen in the photo is a male from the Santa Rosa River collected at the 31 m site. Scale bar is 10mm.
(TIF)

S2 Fig Standard length variation among samples. E = Esmeraldas River, J = Jubones River, SR = Santa Rosa River, and GO = Guayas and other small neighboring drainages.
(TIF)

S3 Fig Water temperature at sampling sites measured during specimen collection. Temperature data for the Esmeraldas and Jubones sites were measured in July 2014 and for the Santa Rosa River in July 2013.
(TIF)

S4 Fig Elevational gradients for the sampling sites in the Esmeraldas, Jubones and Santa Rosa rivers. Distances from ocean are approximate and were measured along river courses in Google Maps.
(TIF)

S1 Table Water quality data for the sampling sites in the Esmeraldas, Jubones, and Santa Rosa river drainages. (DOC)

S2 Table Haplotype frequency of COI mtDNA gene for *Rhoadsia* samples. N is the number of specimens per sample.
(DOC)

S3 Table S7 intron allele frequencies for *Rhoadsia* samples. (DOC)

S4 Table Pairwise F_ST_ values between samples. COI gene below diagonal, S7i2 gene above diagonal. Significant F_ST_ values in bold with asterisks indicating the level of significance.
(DOC)

S1 Appendix Catalog numbers for voucher specimens deposited in museums listed by drainage and site specimen number listed in parenthesis for sites with more than one lot examined. CAS: California Academy of Sciences, FMNH: Field Museum of Natural History, MECN: Museo Ecuatoriano de Ciencias Naturales (Ecuador), MUGT: Museo de Ciencias Naturales de la Universidad de Guayaquil (Ecuador).
(DOC)
