# Supplementary figures and images for: Parallel body shape divergence in the Neotropical fish genus Rhoadsia (Teleostei: Characidae) along elevational gradients of the western slopes of the Ecuadorian Andes
Source: PLoS One. 2017 Jun 28;12(6):e0179432. doi: 10.1371/journal.pone.0179432 (PMC5489170; doi:10.1371/journal.pone.0179432)

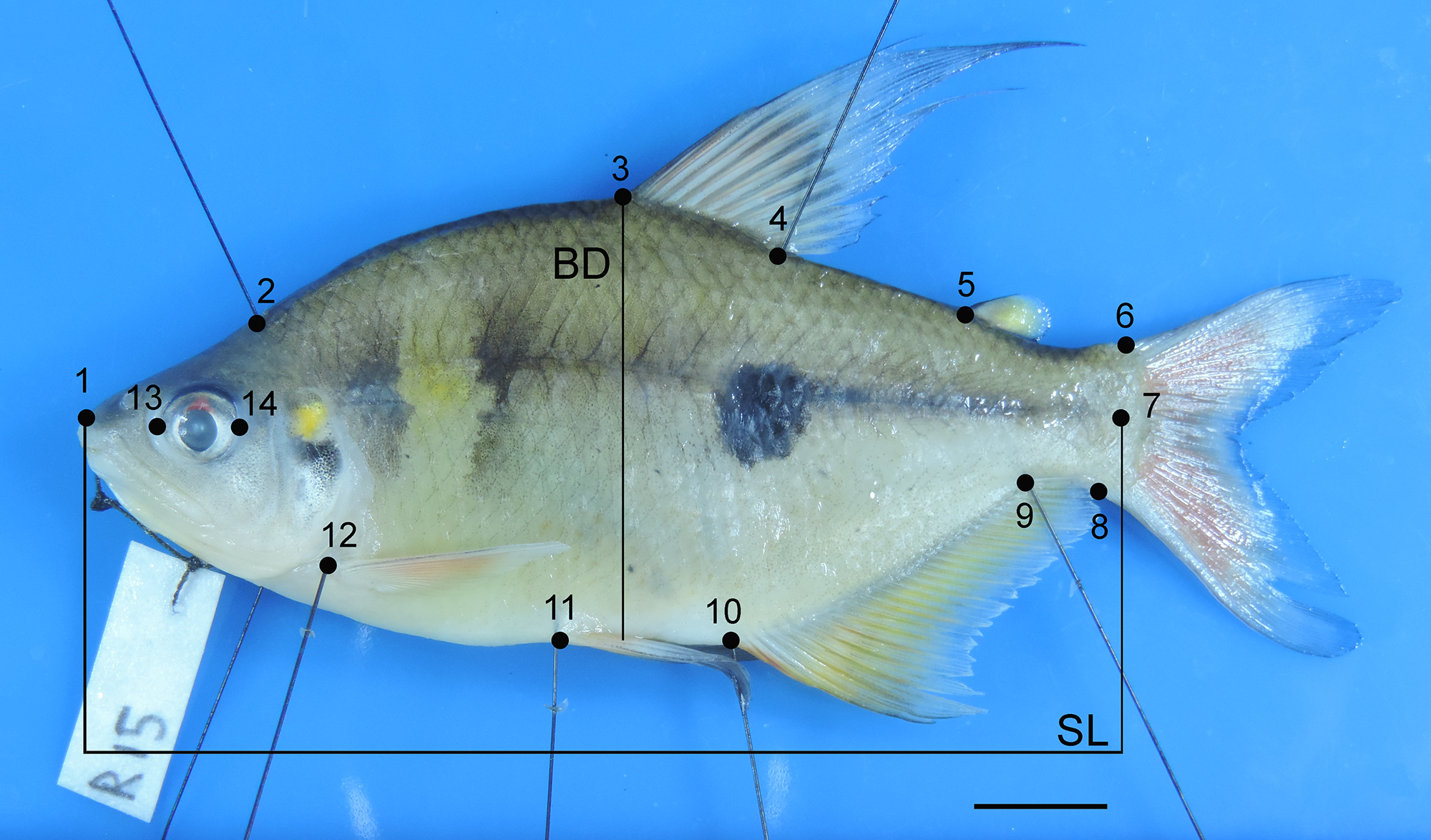

Supplement: S1 Fig — Insect pins were used to mark some of the landmarks that were difficult to see from a lateral perspective. SL is standard length and BD is body depth. The specimen in the photo is a male from the Santa Rosa River collected at the 31 m site. Scale bar is 10mm. (TIF) [file pone.0179432.s001.tif]

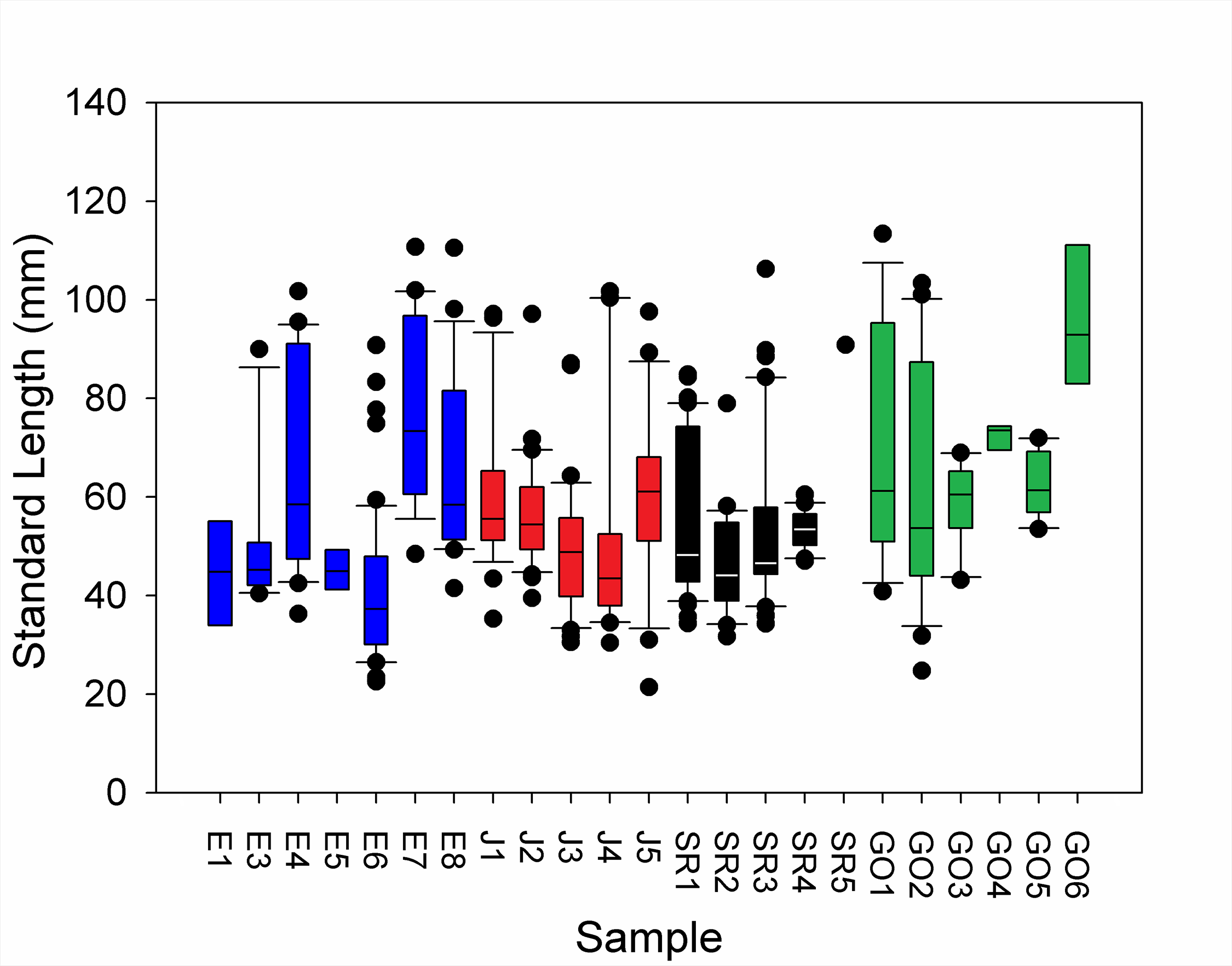

Supplement: S2 Fig — E = Esmeraldas River, J = Jubones River, SR = Santa Rosa River, and GO = Guayas and other small neighboring drainages. (TIF) [file pone.0179432.s002.tif]

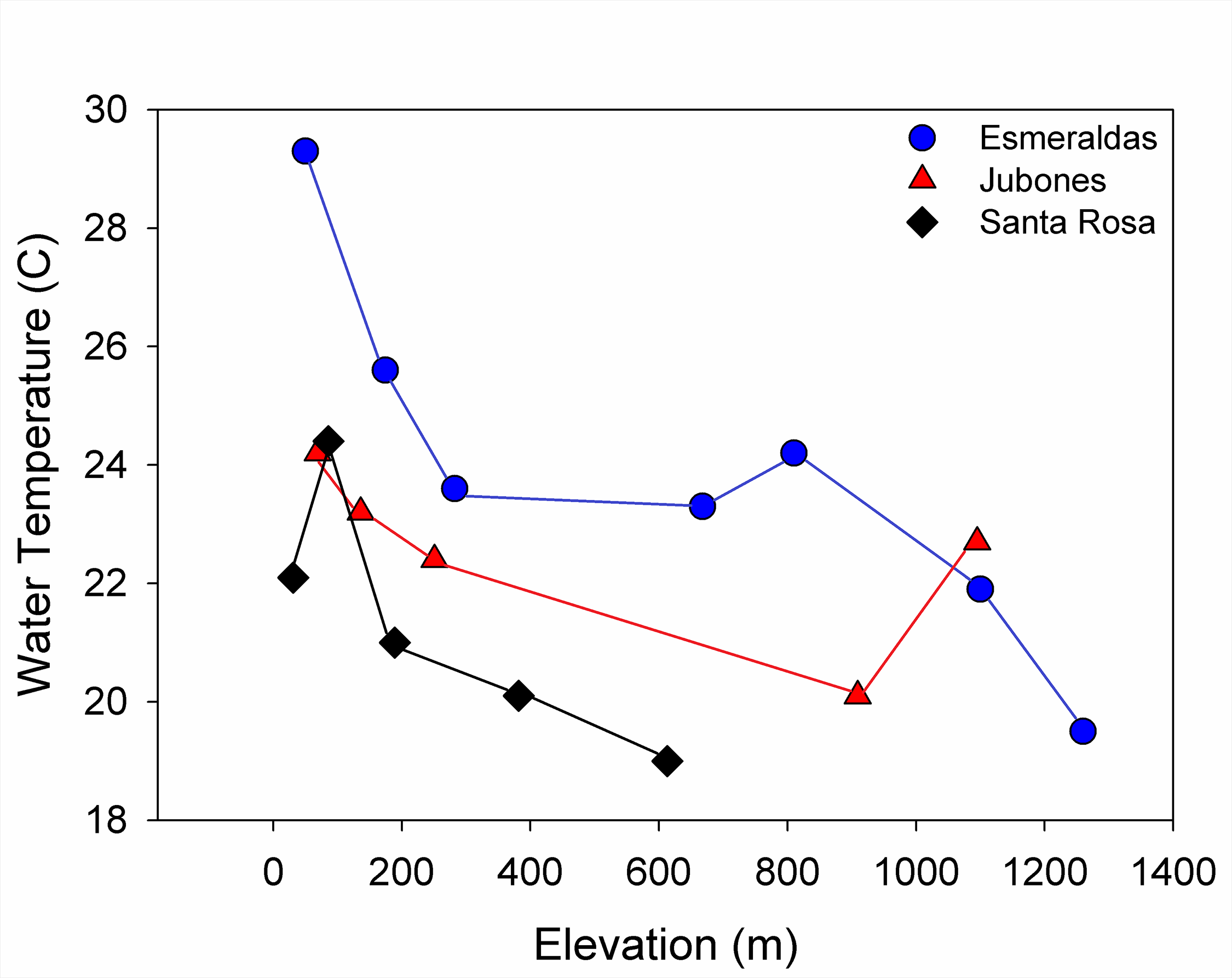

Supplement: S3 Fig — Temperature data for the Esmeraldas and Jubones sites were measured in July 2014 and for the Santa Rosa River in July 2013. (TIF) [file pone.0179432.s003.tif]

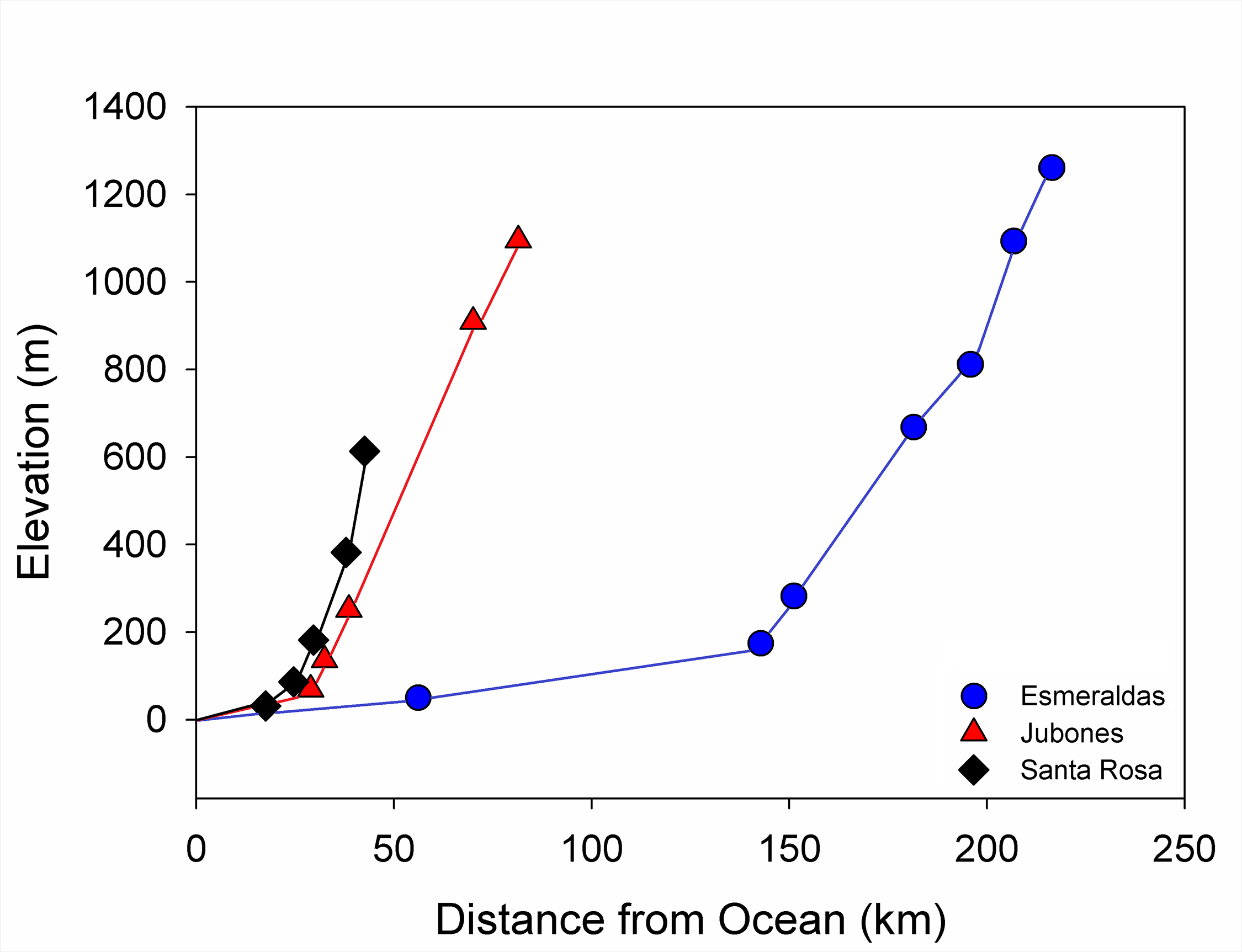

Supplement: S4 Fig — Distances from ocean are approximate and were measured along river courses in Google Maps. (TIF) [file pone.0179432.s004.tif]
