# Supplementary material for: Parallel body shape divergence in the Neotropical fish genus Rhoadsia (Teleostei: Characidae) along elevational gradients of the western slopes of the Ecuadorian Andes
Source: PLoS One. 2017 Jun 28;12(6):e0179432. doi: 10.1371/journal.pone.0179432 (PMC5489170; doi:10.1371/journal.pone.0179432)
Supplement: S1 Table — (DOC) [file pone.0179432.s005.doc]

S1 Table. Water quality data for the sampling sites in the Esmeraldas, Jubones, and Santa Rosa river drainages.

Site Elevation (m) Temp (˚C) Spec Cond O_2_ (mg/ml) pH Wat Vel (m/s)

Esmeraldas

E1 50 29.3 498.0 9.1 7.8 -

E3 174 25.6 40.1 10.1 8.0 0.7

E4 282 23.6 56.5 8.8 7.6 0.4

E5 810 24.2 128.4 7.4 7.9 0.1

E6 1260 19.5 134.4 9.3 7.9 0.5

E7 668 23.3 82.8 8.9 7.9 0.4

E8 1100 21.9 90.0 9.2 8.0 1.0

Jubones

J1 69 24.2 86.5 9.0 7.9 0.2

J2 136 23.2 45.7 9.6 8.1 0.7

J3 251 22.4 63.9 9.8 7.9 0.2

J4 909 20.1 - 9.8 8.4 0.2

J5 1095 22.7 272.9 8.7 8.0 0.2

Santa Rosa*

SR1 31 22.1 150.3 8.5 - 0.3

SR2 86 24.4 132.4 8.9 - 0.4

SR3 189 21.0 113.9 8.3 - 0.9

SR4 382 20.1 76.2 8.3 - 0.4

SR5 613 19.0 48.0 8.3 - 0.3

Data for the Esmaraldas and Jubones rivers are based on measures taken in July 2014. *Data for the Santa Rosa River are based on average measures taken in July 2013 (Aguirre et al., 2016).
