# Supplementary material for: Parallel body shape divergence in the Neotropical fish genus Rhoadsia (Teleostei: Characidae) along elevational gradients of the western slopes of the Ecuadorian Andes
Source: PLoS One. 2017 Jun 28;12(6):e0179432. doi: 10.1371/journal.pone.0179432 (PMC5489170; doi:10.1371/journal.pone.0179432)
Supplement: S2 Table — N is the number of specimens per sample. (DOC) [file pone.0179432.s006.doc]

S2 Table. Haplotype frequency of COI mtDNA gene for *Rhoadsia* samples. N is the number of specimens per sample.

Site N H_1_ H_2_ H_3_ H_4_ H_5_ H_6_ H_7_ H_8_ H_9_ H_10_ H_11_ H_12_ H_13_

E01 6 - - 6 - - - - - - - - - -

E03 10 - - 7 - - - - - - - - 2 1

E04 8 - - 8 - - - - - - - - - -

E05 8 - - 8 - - - - - - - - - -

E06 13 - - 12 1 - - - - - - - - -

E07 8 - - 7 - - - - 1 - - - - -

E08 8 5 - 2 - - 1 - - - - - - -

J01 9 - - - 1 2 4 - - 2 - - - -

J03 6 - - - 2 1 - 2 - - 1 - - -

J04 8 - - - - - 3 1 - - 3 1 - -

J05 10 - - - - - - 5 - - 5 - - -

G01 15 13 - - - - - 2 - - - - - -

G02 16 11 1 1 3 - - - - - - - - -

SR 16 - - - 4 11 1 - - - - - - -

Total 141 29 1 51 11 14 9 10 1 2 9 1 2 1

Freq 0.21 0.01 0.36 0.08 0.10 0.06 0.07 0.01 0.01 0.06 0.01 0.01 0.01
