# Supplementary material for: Parallel body shape divergence in the Neotropical fish genus Rhoadsia (Teleostei: Characidae) along elevational gradients of the western slopes of the Ecuadorian Andes
Source: PLoS One. 2017 Jun 28;12(6):e0179432. doi: 10.1371/journal.pone.0179432 (PMC5489170; doi:10.1371/journal.pone.0179432)
Supplement: S3 Table — (DOC) [file pone.0179432.s007.doc]

S3 Table. S7 intron allele frequencies for *Rhoadsia* samples.

Site A1 A2 A3 A4 A5 Sum

E01 - 2 6 2 - 10

E03 14 - - - - 14

E04 16 - - - - 16

E06 10 5 1 - - 16

E07 3 7 2 - - 12

E08 16 - - - - 16

J01 - 1 8 - 5 14

J03 15 1 - - - 16

J05 - 4 12 - - 16

G01 8 - - 6 - 14

G02 - 8 6 1 1 16

SR 1 5 10 - - 16

N 83 33 45 9 6 176

Freq 0.47 0.19 0.26 0.05 0.03
