# Supplementary material for: Parallel body shape divergence in the Neotropical fish genus Rhoadsia (Teleostei: Characidae) along elevational gradients of the western slopes of the Ecuadorian Andes
Source: PLoS One. 2017 Jun 28;12(6):e0179432. doi: 10.1371/journal.pone.0179432 (PMC5489170; doi:10.1371/journal.pone.0179432)
Supplement: S4 Table — COI gene below diagonal, S7i2 gene above diagonal. Significant FST values in bold with asterisks indicating the level of significance. (DOC) [file pone.0179432.s008.doc]

S4 Table. Pairwise F_ST_ values between samples. COI gene below diagonal, Si72 gene above diagonal. Significant F_ST_ values in bold with asterisks indicating the level of significance.

E1 E3 E4 E5 E6 E7 E8 J1 J3 J4 J5 GO1 GO2 SR

E1 - **0.851***** **0.861***** X **0.584*** 0.381*** 0.861*** 0.160* 0.816***** NA 0.001 **0.251**** 0.064 0.031

E3 0.091 - 0.000 X **0.267* 0.634***** 0.000 **0.861***** 0.000 NA **0.878*** 0.385*** 0.746*** 0.788*****

E4 0.000 0.134 - X **0.286* 0.654***** 0.000 **0.870***** 0.000 NA **0.886*** 0.406*** 0.759*** 0.800*****

E5 0.000 0.134 0.000 - NA NA NA NA NA NA NA NA NA NA

E6 0.000 0.163 0.000 0.000 - 0.13491 **0.286*** **0.660***** 0.172 NA **0.619*** 0.189* 0.437*** 0.487*****

E7 0.000 0.112 0.000 0.000 0.013 - **0.654***** **0.517*** 0.559***** NA **0.406*** 0.182* 0.144 0.229***

E8 **0.388* 0.364*** 0.440* 0.440* 0.490*** 0.408***** - **0.869***** 0.000 NA **0.886*** 0.406* 0.759*** 0.800*****

J1 **0.638*** 0.620*** 0.675*** 0.675*** 0.688*** 0.644*** 0.526**** - **0.836***** NA **0.197** 0.421*** 0.240** 0.223****

J3 **0.600*** 0.580*** 0.650*** 0.650*** 0.669*** 0.609*** 0.480** 0.182*** - NA **0.844*** 0.358*** 0.709*** 0.751*****

J4 **0.714*** 0.696*** 0.747*** 0.747*** 0.767*** 0.720*** 0.595*** 0.298***** 0.046 - NA NA NA NA

J5 **0.900*** 0.843*** 0.911*** 0.911*** 0.904*** 0.884*** 0.757*** 0.667***** **0.329*** 0.220 - **0.367***** 0.053 0.000

GO1 **0.614*** 0.579*** 0.641*** 0.641*** 0.669*** 0.620***** 0.009 **0.676*** 0.632*** 0.705*** 0.802***** - **0.254** 0.277*****

GO2 **0.495*** 0.471*** 0.526*** 0.526*** 0.556*** 0.507**** 0.000 **0.626*** 0.591*** 0.686*** 0.790***** 0.005 - 0.000

SR **0.794*** 0.737*** 0.811*** 0.811*** 0.795*** 0.779*** 0.664***** 0.132 **0.452** 0.601*** 0.832*** 0.762*** 0.707***** -

* P < 0.05, ** P < 0.01, *** P < 0.001
