# Supplementary material for: Parallel body shape divergence in the Neotropical fish genus Rhoadsia (Teleostei: Characidae) along elevational gradients of the western slopes of the Ecuadorian Andes
Source: PLoS One. 2017 Jun 28;12(6):e0179432. doi: 10.1371/journal.pone.0179432 (PMC5489170; doi:10.1371/journal.pone.0179432)
Supplement: S1 Appendix — CAS: California Academy of Sciences, FMNH: Field Museum of Natural History, MECN: Museo Ecuatoriano de Ciencias Naturales (Ecuador), MUGT: Museo de Ciencias Naturales de la Universidad de Guayaquil (Ecuador). (DOC) [file pone.0179432.s009.doc]

S1 Appendix. Catalog numbers for voucher specimens deposited in museums listed by drainage and site specimen number listed in parenthesis for sites with more than one lot examined. CAS: California Academy of Sciences, FMNH: Field Museum of Natural History, MECN: Museo Ecuatoriano de Ciencias Naturales (Ecuador), MUGT: Museo de Ciencias Naturales de la Universidad de Guayaquil (Ecuador).

-Esmeraldas: E1: FMNH-126210; E3: FMNH-126213; E4: FMNH-126214; E5: MECN-DP-3350; E6: CAS 32457 (n=19), FMNH-126212 (n=37); E7: FMNH-126211.; E8: MECN-DP-3349; Guayas-Other: GO1: MUGT P-0226 (n=3), MUGT P-0380 (n=5), MUGT P-0404 (n=7); GO2: FMNH-79077 (n=10), FMNH-79080 (n=5), FMNH-71867 (n=9); GO3: MUGT P-0416; GO4: MUGT P-198; GO5: MUGT P-0300; GO6: MUGT P-0011. Jubones: J1: FMNH-126318, MECN-DP-3347; J2: MECN-DP-3346; J3: FMNH-126319; J4: MECN-DP-3347; J5: FMNH-126289. Santa Rosa: SR1: FMNH-122407 (n=44), MECN-DP-2647 (n=10); SR2: MECN-DP-2626; SR3: MECN-DP-2641; SR4: FMNH-122431 (n=6), MECN-DP-2647 (n=8), SR5: FMNH-122423.
